# Limits on the capacity of an ectotherm to buffer temperature extremes through the construction of a central refuge

**DOI:** 10.1007/s00442-026-05959-6

**Published:** 2026-08-20

**Authors:** Terry J. Ord, Katrina Blazek

**Affiliations:** 1 Evolution and Ecology Research Centre, School of Biological, Earth and Environmental Sciences, University of New South Wales, Kensington, NSW 2052 Australia; 2 School of Population Health, University of New South Wales, Kensington, NSW 2052 Australia

**Keywords:** Climate change, Long-term field study, Nest conditions, Nest depth, Refuge insulation

## Abstract

Competition, lack of space, and prolonged environmental change can lead animals to build refuges in areas that are (or become) exposed or otherwise offer poor protection. This is especially problematic for species that invest heavily in refuge construction and reside in that refuge long-term. We examined the consequences of refuge position in an iconic central place forager, the Australian meat ant (*Iridomyrmex purpureus*). Colonies excavate massive ground nests out of soil and can remain in those nests for a decade or more. We monitored the external and internal temperature of nests for over two years. These data were combined with a phylogenetic analysis of optimal brood incubation temperatures compiled from the literature to infer the probable incubation temperature for this species. Focussing on summertime extremes, our field data across consecutive years showed the spatial position of meat ant nests had critical impacts on the internal conditions of nests. In most instances, colonies would probably be able to protect broods by actively moving broods to different depths within the nest. However, this thermoregulation would unlikely fully compensate for the extreme temperatures experienced by the most exposed nest in our study. This exposed nest has in turn suffered a sustained decline in colony size over the last decade. Even for a central place forager believed to be resilient to environmental degradation, refuges could not protect against prolonged environmental change, with likely tangible impacts on fitness.

**Supplementary Information:**

The online version contains supplementary material available at https://doi.org/10.1007/s00442-026-05959-6.

## Introduction

Many animals invest considerable time and energy in building a refuge. These include the excavation of burrows (insects: Pocock 1901; crustaceans: Christy 1982; birds: Smith and Conway 2007; mammals: Evans 2008) and the construction of nests (insects: Franks et al. 2006; birds: Newton 1994; mammals: Lutermann et al. 2010) that serve as shelters against environmental conditions and protection against enemies (e.g., predators). Refuges subsequently improve survival but can also provide benefits for reproduction. In particular, the buffer against fluctuations in external environmental conditions provided by a refuge allows internal conditions to be regulated for the incubation of eggs and development of young (Li et al. 2017; Colombo et al. 2024; Corimanya et al. 2024). While the way a refuge is constructed—e.g., the materials used—can dictate how well internal conditions can be maintained (Hilton et al. 2004; Fast et al. 2010; Breen et al. 2021), the location chosen for a burrow or nest is also influential (Hilton et al. 2004; Lopez-Luna et al. 2020; Ord and Blazek 2025). Obtaining appropriate construction materials or the optimal placement of a refuge are not always possible because resources or space are scarce or limited by competition (Newton 1994; Charter et al. 2016; Ord 2023; Svensson and Kvarnemo 2023). Nevertheless, in many cases an animal might be able to mitigate suboptimal placement of a refuge through the materials used to construct the refuge itself, or vice versa. The capacity for such mitigation would not only improve the survival and reproductive success of individual refuge builders, but potentially the capacity of a population as a whole to resist local environmental adversity. The resilience of populations to extreme environmental conditions is of special concern given the increase in severity and frequency of such events from the climate crisis (Rahmstorf and Coumou 2011; Coumou and Rahmstorf 2012; Screen and Simmonds 2014; Stevenson et al. 2022). There is a particular need for understanding how and where refuges are constructed, as this might improve (or limit) population resilience, especially in ectotherms who are acutely sensitive to fluctuations in environmental temperature (Nowrouzi et al. 2019; Couper et al. 2021; Parr and Bishop 2022).

Many ectotherms rely on the construction of burrows or nests as a homebase or central place to return to following bouts of foraging. Ants are classic examples of such ‘central place foragers’ and build nests that not only shelter the colony but are used to moderate conditions for brood development (e.g., see Table S1). These nests can be constructed out of the living bodies of workers themselves (e.g., army ants: Baudier et al. 2018), deadwood (Roces and Nunez 1995; Kipyatkov and Lopatina 2002; Mezger and Pfeiffer 2010), sticks (Kipyatkov and Lopatina 2002) or leaves (Kipyatkov and Lopatina 2002). However, many species excavate nests out of soil on the ground (e.g., Murdock and Tschinkel 2015; Baudier and O’Donnell 2016; Camargo et al. 2016). The conditions inside these soil nests are buffered from external temperatures to some degree because of the insulating properties of the soil. The colony can further control the internal temperatures experienced by broods by physically moving the position of the brood inside the nest (Roces and Nunez 1989, 1995; Anderson and Munger 2003; Pranschke and Hooper-Bui 2003). Nevertheless, the location of the nest within the environment is still likely to be an important influence on the internal conditions of a nest. In particular, the extent to which the insulating properties of soil or repositioning of the brood within the nest can adequately compensate for poor nest placement is unclear. At a broad level, ant species that excavate soil nests on the ground are assumed to be less prone to adverse changes in local conditions than species constructing arboreal nests from leaves (Parr and Bishop 2022). But data on the resilience of soil nesting species to extreme changes in environmental conditions, and how nest location within the landscape might mediate that resilience, is rare.

Nevertheless, there is some evidence that the survival of long-lived ant colonies that excavate soil nests are vulnerable to long-term changes in the surrounding environment (Ord 2023; Ord and Blazek 2025). The Australian meat ant (*Iridomyrmex purpureus*) is an iconic central place forager that has a conspicuous and dominant presence in the landscape of many environments in southeastern regions of the country (Anderson and Patel 1994; Anderson and Majer 2004). Meat ants excavate ground nests out of soil and decorate the surface of the nest with tiny pebbles (or, less frequently, short thin twigs). The precise function of this decoration is unknown but has been speculated to improve the thermal regulatory conditions of the nest (Ettershank 1971). Nests are often large, housing millions of workers (Greaves and Hughes 1974) and can remain in place for decades (Greenslade 1975a; Ord 2023). This permanency comes at a cost. Although meat ants can move a colony to a new location by budding (Holldobler and Carlin 1985), they do so rarely (Greaves and Hughes 1974; Greensland 1975a, 1975b; Ord 2023). For example, we have been monitoring a large population of meat ant colonies in the New South Wales Central Tablelands for over a decade (since 2010). The frequency of new nests established in this population is low (< 0.2 nest per hectare; Ord 2023) but escalates substantially with deteriorating environmental conditions (> 0.8 nest per hectare; Ord 2023). Our study area experienced the worst drought on record (2017 through to early 2020, a drought that impacted most of southeastern Australia; bom.gov.au) and many colonies with nests positioned in locations that initially balanced sun exposure became exposed because of vegetation dieback. Colonies budded satellite nests in an apparent attempt to shift the colony to a new, more favourable location (Ord 2023). The success of this strategy was mixed. Many colonies were still observed going extinct (Ord 2023; Ord and Blazek 2025). Although there was likely a drop in food resources because of the drought, nests in locations prior to drought that were close to the edge of the optimal thermal niche envelop for this species (quantified by Ord and Blazek 2025) were observed to dramatically decline in size once drought set in (Ord 2023; Ord and Blazek 2025). This implies the insulating properties of constructing a nest in soil wasn’t enough to compensate for poor nest location, and that behavioural buffers (e.g., thermal regulation of broods inside the nest or more drastic measures such as colony relocation) could not adequately compensate for the deterioration in environmental conditions.

This current study builds on past findings to better resolve the likely key causal mechanism underlying colony survival as it relates to nest position. To this end, we strategically selected three nests representing the broad spectrum of conditions experienced by meat ant colonies in this study area. Earlier study was based on tracking the survival of all 111 meat ant nests found in the study area over 8 + years (Ord 2023). This was coupled with observations of worker behaviour outside of nests for 11 colonies and monitoring of nest surface temperatures for 6 (of the 11) colonises for almost 3 years (Ord and Blazek 2025). These nest subsets were specifically selected (of the 111 nests) to represent the range of nest positions and subsequent surface conditions experienced by colonies over the broader distribution of nests at this site (Ord 2023). In this new study, we narrowed our attention to 3 (of the 11) colonies to intensively monitor temperature conditions inside meat ant nests, and explicitly as a function of external conditions experienced because of nest position. Previous study found little difference in worker behaviour outside of the nest across colonies (11 colonies studied; Ord and Blazek 2025), but documented large differences in nest surface temperatures reflective of nest position (6 colonies; Ord and Blazek 2025). We used these data to identify the two nests likely to exhibit the largest differences in nest surface temperatures across meat ant colonies in this study area (‘exposed’ vs. ‘sheltered’ nests), and supplemented with a third, intermediate nest between these extremes (‘moderate’ nest).

By fitting internal temperature loggers inside these three nest and then monitoring conditions for at least two years, the objective of the current study was to determine the extent external fluctuations in temperature were buffered by: (1) the insulating properties of excavating nests out of soil and the general architecture of how meat ants construct those nests; and (2) the potential for workers to physically move broods deeper into a nest to further mitigate deteriorating conditions. These data were combined with information on the temperature conditions of the surrounding soil and those experienced at the surface of the nest. The incubation temperature of meat ant broods was unknown. We therefore compiled available published information on brood incubation temperatures for other ant species from the literature and used phylogenetic comparative methods to compute the likely optimal temperature range for brood incubation for meat ants. This information was combined with other data from the literature on summertime nest temperatures recorded at varying depths inside ant nests. Together, these data were used to explore how the ideal depth for incubating broods might fluctuate inside the nest over the course of a typical summer day. We then discuss a general mechanistic explanation of how changes in external environmental conditions might impact colony survival in meat ants, under the assumption that the three target nests investigated provide a reasonable reflection of the range of conditions experienced by colonies in this study area.

## Materials and methods

### Study system

The meat ant population occurs on a private property near the locality of Wollar in the New South Wales Central Tablelands. All nests have been mapped and permanently labelled with metal tags and systematically monitored since 2015 (and informally since 2010). Most nests are clustered along the boundary of an open grassland and eucalyptus woodland. Nest position relative to this grassland-woodland boundary, as well as to a surrounding raised escarpment, determines the amount of shade received by a nest. Some nests are positioned in locations that expose the nest to high solar radiation or long periods of shade, with both conditions of excessive sun or shade linked to colony decline and death (Ord 2023; Ord and Blazek 2025).

Monitoring consisted of an exhaustive annual survey of all nests at the site. This survey recorded a nest’s spatial position using a Global Positioning System (GPS), the number of entrance holes (which correlates with nest size—Greaves and Hughes 1974; Ord 2023–and can be used to monitor nest growth and decline—Ord 2023; Ord and Blazek 2025), and the presence of foraging trails and connection trails between nests. Colonies are usually housed in a single nest, but some are distributed amongst as many as seven nests (Ord 2023). In addition to this annual survey (Ord 2023), the surface conditions of the nests of six colonies were continuously monitored for almost 3 years (Ord and Blazek 2025). These nests were selected because of their spatial position relative to the grassland-woodland boundary and surrounding escarpment, such that these nests encompassed the likely range of temperature conditions experienced by most meat ant colonies at this study site.

Using this extensive library of data (reported in Ord 2023 and Ord and Blazek (2025), we targeted three nests that we could confirm belonged to separate colonies and were housed exclusively in this single nest. More specifically, though, these three nests were targeted because earlier study of these and other nests (Ord and Blazek 2025) implicated these three nests could be expected to reflect the full range of conditions experienced by colonies at this site. Nest XI was the most exposed nest and the first nest at the site to receive morning sun and the last to receive afternoon shade (nest XI: ‘exposed’ nest), while nest XCIII was the last to receive morning sun and the first to receive afternoon shade (nest XCIII: ‘sheltered’ nest). Nest III was anticipated to have an intermediate level of exposure between these two extremes (nest III: ‘moderate’ nest). At the start of the study, the size of these nests were: nest XI had 58 entrance holes (approximately ~ 770,000 workers); nest III had 35 entrance holes (~ 450,000 workers); and nest XCIII had 37 entrance holes (~ 480,000 workers; based on formulae described in Ord 2023).

### Measuring nest conditions in situ

All temperature recordings were made at 30-minute intervals using TinyTag Plus 2 data loggers (TGP-4020) fitted with standard metal-cased temperature probes (PB-4720). Loggers were housed inside Steveson Type Screens (ACS-5050) positioned on posts pitched into the ground adjacent to the nest and at a height to ensure probe cables remained elevated off the nest. Probe cables were fitted inside a thick rubber garden hose, which was in turn wrapped with fencing wire in attempt to armour the cable against wildlife chewing. Temperature was recorded at the tip of the probe, with the manufacturer’s insulated cabling and the secondary housing of the cable inside the rubber hose ensuring recordings were not influenced by the temperature of the cable itself.

Two sets of temperature probes and data loggers were placed on each of the three nests. The first probe was housed inside a custom-made PVC sleave to ensure the probe’s metal casing was not exposed to direct sun (which would impact temperature recordings). This sleave was anchored in place with metal spikes so the tip of the probe recorded temperature approximately 1 cm above the surface of the nest. In this suspended position, the probe effectively recorded the temperature that would be experienced by a meat ant worker walking on the surface of the nest. The second probe was pushed directly into the centre of the nest to a depth of 7 cm. This depth roughly corresponded to the fourth tier of chambers from the nest surface (e.g., Figure S1), and is a depth where eggs and larvae have been observed when nests have been freshly predated (TJO, personal observation). Probes remained in place from November 5, 2018 through to March 14, 2021. This period included 16 months of severe drought (2018–2020) and 12 months post-drought (2020–2021).

For two of the three nests (Nest XI – the most ‘exposed’ nest – and XCIII – the least exposed ‘sheltered’ nest), two additional sets of temperature probes and data loggers were positioned 3–4 m adjacent to the nest. The position of these ‘off-nest’ probes were carefully selected so the surface probe was suspended approximately 1 cm above an area free of ground vegetation. The temperature recorded by this probe was representative of what a meat ant worker would experience when foraging away from the nest. The second probe was pushed into the ground soil to a depth of 7 cm within a few centimetres of the off-nest surface probe. The temperature recorded by this probe was used to measure the insulation properties of soil in the absence of any influence of nest architecture on below-ground temperature. Both ‘off-nest’ probes were positioned relative to the shifting area of shade over the course of the day to match the changes in exposure of probes placed directly on the adjacent nest. That is, any difference in temperatures inside the nest at 7 cm depth compared to off the nest at 7 cm depth would specifically reflect the influence of the architecture of the nest in buffering external temperature fluctuations beyond simply the insulating properties of the soil. Probes remained in place from July 9, 2020 through to March 14, 2021. This period covered only the post-drought months.

### Optimal incubation temperatures and nest temperatures at depth

ISI Web of Science was used to identify studies where authors had determined the preferred incubation temperatures of ant broods, either experimentally in the lab or through destructive excavation of nests in the field. Search terms consisted of ‘ant*’ and ‘nest temperature*’ searched with one of the following: ‘egg*’, ‘brood’, ‘larv*’, ‘ovipositor*’, ‘develop*’ or ‘life history’. These searches were combined with ‘develop*’ and ‘meat ant’ as well as ‘develop*’ and ‘Iridomyrnex’. Terms were searched in all fields, all years and all categories. In total, 361 abstracts were reviewed, with 93 downloaded for closer examination. No incubation temperatures were found for the meat ant or any other species of the same genus. We therefore used the data from other species to estimate the probably incubation temperature of broods for meat ants using phylogenetic statistical methods (see ‘Phylogenetic inferences of meat ant incubation temperature’).

Given our internal temperature monitoring was limited to a depth of 7 cm, we took the opportunity while reviewing the 93 papers above to compile data from eight papers that reported internal nest temperature as a function of depth in summer. This was done to provide a general view of how nest temperature changes with depth, which was used to inform the extent to which we could extrapolate beyond a depth of 7 cm for our nests. We included an additional study on *I. purpureus* (Ettershank 1971) as a key further benchmark. This study reported nest temperatures for a single nest in summer and spring using two methods. In an initial pilot study conducted over two days in summer, mercury-in-glass thermometers were positioned at the nest surface and inside the nest at a depth of 10 cm (Ettershank 1971). In the main study conducted over four days in spring, more accurate electric temperature probes were positioned inside the nest at depths of 3 cm, 8 cm, 23 cm and 38 cm (Ettershank 1971). Air temperature was also recorded in this springtime study at a height of 5 cm above the nest. We did not include these data because the pilot showed temperature recorded 5 cm above the nest was 3–9 °C cooler than the temperature recorded at the nest surface (Ettershank 1971).

Data and sources are reported in Tables S1 and S2.

### Statistical analyses

#### Nest conditions of meat ant nests

Nest probes were routinely displaced by wildlife (wombats, kangaroos, choughs and magpies; TJO personal observation), who would either accidentally dislodge probes from the nest or ground by tripping over the probe cable, or by the action of attempting to chew probe cables. Periods where probes had been knocked out of position were identified through regular site visits and any subsequent periods of interruption were excluded from the data set. For statistical analyses, complete overlapping data was required to compare temperatures between surface and internal probes for a given nest (probes positioned 1 cm above the nest surface versus 7 cm below the nest surface), as well as probes positioned on different nests (nests XI, III and XCIII). For on-nest analyses of all three nests, we focussed on the longest period of overlap occurring for six probes during peak summer, which corresponds to the period typified by the most extreme fluctuations in nest temperature (Ord and Blazek 2025). This was a three-month period from January 1, 2019 through to March 31, 2019. For on-vs-off nest analyses of nests XI and XCIII, the longest period of overlap for all probes during peak summer was January 1–31, 2021. In summary, comparisons among the three nests of surface and internal probes (+ 1 cm and − 7 cm) were made over a 12-week period during summer, while comparisons of two nests fitted with surface and internal probes (+ 1 cm and − 7 cm) both on and off the nest were made over a four-week period. These periods were representative of summer fluctuations in temperature across the full monitoring period of 2018–2021 (see Figure S1).

**Fig. 1:**
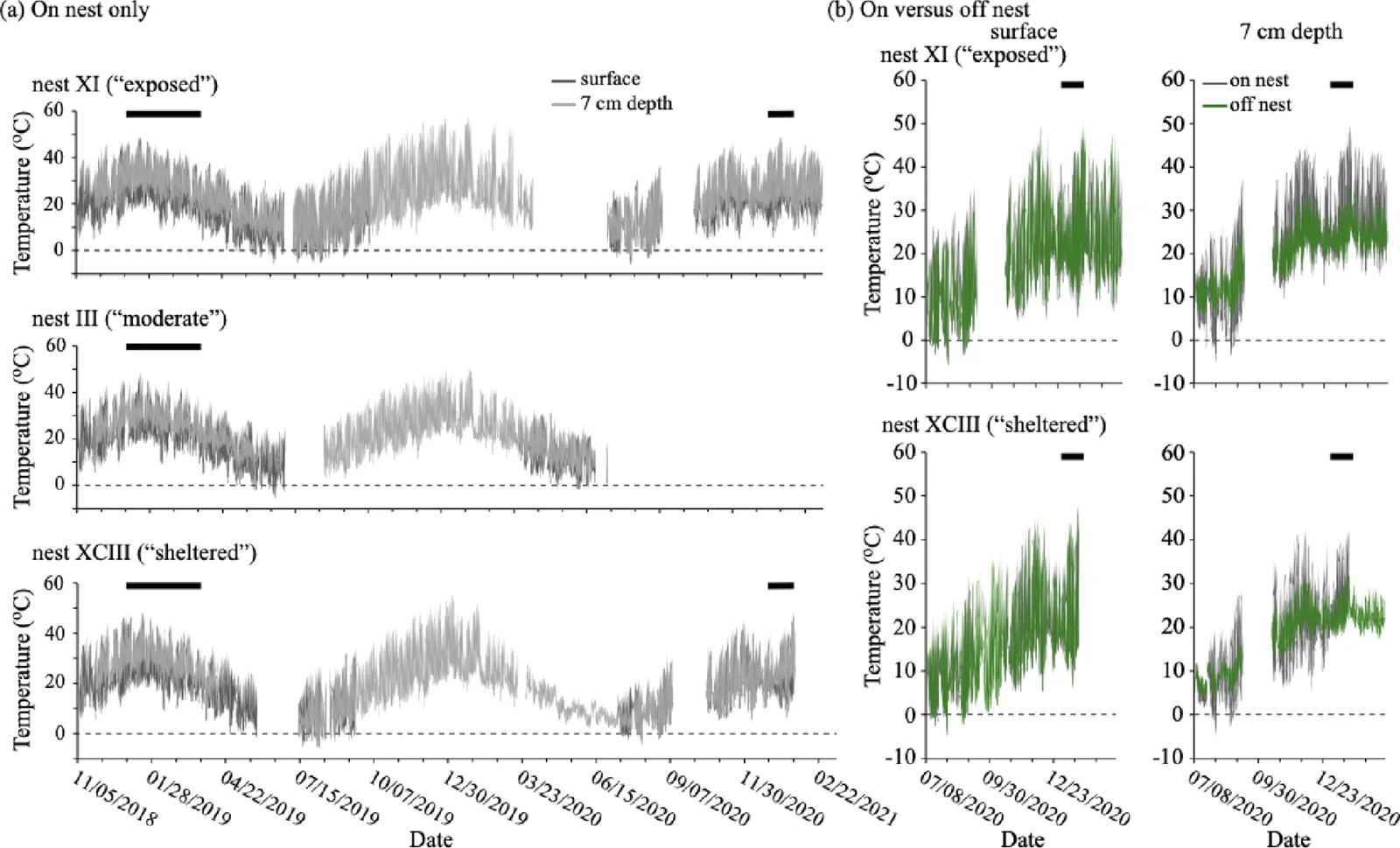
Oscillations in half-hourly temperature (**a**) recorded 1 above the nest surface and at a depth of 7 cm inside the nest, and (**b**) on versus off the nest. Gaps in the data reflect instances where probes had been knocked off the nest or severed from data loggers by cables being bitten through by wildlife (birds, wombat and kangaroo). The horizontal bars above each plot correspond to the overlapping periods used in analyses reported in Tables 1 and 2, respectively. These data are also shown in Figs. 2a and 3a

All statistical analyses were applied using R version 4.3.1 (R Development Core Team, R Foundation for Statistical Computing, Vienna). Analyses included time (in 30-minute intervals) as the primary predictor variable for both the 12-week (on-nest, three nest comparison) and 4-week data sets (on- and off-nest, two nest comparison) and were conducted in the same manner as follows. The maximum and minimum daily temperature were extracted and analysed by fitting generalized additive mixed models using the ‘mgca’ package version 1.9-1 (Wood 2011, 2017). Initial exploration identified a spline smoothing factor of k = 5 as a reasonable knot number, with fitting diagnostics checked using the ‘gam.check’ function. Three models were then applied to assess the level of support for random effects of the probe recording data using AIC: (i) a random intercept and random slope model; (ii) a random intercept only model; and (iii) no random effect model. A random intercept model was found to be the best supported model for all but one model set (see Tables S3 and S4). In this last instance, both a random intercept model and a no random effect model comparing minimum daily temperatures across three nests over 12-weeks had equivalent support (Table S3). We subsequently selected a random intercept model for this analysis to be consistent across analyses and because a random intercept for the probe recording data made biological sense. All models were then fit with unpenalized splines and diagnostics checked again using the ‘gam.check’ function. For the model comparing maximum daily temperatures on and off the nest, an interaction term between probe location and depth was included (Fig. 1).

To evaluate differences in temperature across nests and probe depths, we applied hierarchical time series clustering based on half-hourly estimates of daytime temperature using the ‘dtwclust’ package version 1.23-1 (Sarda-Espinosa 2024) and a dynamic time warping distance measure. Dendrograms were then used to visualise the computed distance estimates of dissimilarity using the ‘ggplot2’ package version 3.4.2 (Wickham 2016). Exploratory analyses were initially used to identify the number of likely clusters, although varying this number had little impact on results and no effect on conclusions drawn. The main objective of this portion of our analyses was to provide a statistical visualization of the overall similarities/dissimilarities of temperature over time among nests and probe positions (on the nest 1 cm above the surface versus inside the nest at a 7 cm depth).

#### Phylogenetic inferences of meat ant incubation temperature

Data on optimal incubation temperatures compiled from the literature (Table S1) were entered into a phylogenetic comparative analysis to infer the probable incubation temperature for meat ants. We used the phylogeny developed by Nelsen et al. (2018; available from the repository associated with Nelsen et al. 2023). Species not represented in this phylogeny were positioned on the tree based on species belonging to the same genus and occupying a similar geographic location (extrapolated from information obtained from AntWiki.org). In instances where taxa were not identified to species, we arbitrarily positioned taxa within the genus on the phylogeny and set the branch length to the average computed across the species represented on the phylogeny within that genus. Supporting Information provides full details on the phylogenetic positioning of individual taxa.

We used two complementary approaches for inferring the likely optimal incubation temperature for meat ants based on known incubation temperatures compiled from the literature. These two approaches were selected because they offered distinctly different methods of estimation—one through ancestor state reconstruction, the other based on the phylogenetic mean from an evolutionary regression—and assumed alternative modes of how the process of evolution might have proceeded—via a pure Brownian motion (BM) process versus a more complex Ornstein-Uhlenbeck (OU) model. It was not appropriate to select which of these two approaches might provide the better supported inference of incubation temperature (e.g., by comparing AIC estimates) because each method of inference was unique (ancestor state reconstruction versus evolutionary regression). It would be appropriate to compare alternative evolutionary models *within* each approach (e.g., BM versus OU), but the inferred incubation temperatures between the two approaches were effectively equivalent to one another, so doing so would not provide any additional insights. By extension, our inferred incubation temperature for the meat ant can be considered robust to both the phylogenetic method applied (ancestor state reconstruction versus evolutionary regression) and the assumed underlying mode of evolution (BM versus OU).

For the first approach, we applied ancestor state reconstructions using the ‘phytools’ package version 2.3-0 (Revell 2024) and BM model of evolution implemented using maximum likelihood. These phylogenetic reconstructions were then used to infer the optimal incubation temperature of meat ants based on three reconstructions obtained from data of: (i) ‘all species’ regardless of materials used to construct nests or the climate zone species were predominantly found (N_species_ = 59); (ii) ‘temperate species only’ and regardless of what materials species used to construct nests (N_species_ = 7); and (iii) ‘soil nesting species only’ and regardless of the climate zone species were predominantly found (N_species_ = 13). There were not enough data to estimate incubation temperature from both soil nesting and temperate species (N_species_ = 3). We conducted an accuracy assessment of this approach by iteratively removing known incubation temperatures for single species and used phytools to infer its value based on an ancestor state reconstruction of the remaining species in the ‘all species’ data set. This inferred estimate of optimal incubation temperature was subtracted from its known optima and converted to an absolute value, with an average taken across the 59 iterative species removals. We used this average difference as an accuracy index of how well the ancestor state reconstruction inferred the probable optimal incubation temperature for meat ants.

For the second approach, we applied phylogenetic regressions using the ‘phylolm’ package version 2.6.2 (Ho and Ane 2014) and an Ornstein-Uhlenbeck (OU) model of evolution using maximum likelihood. We first explored the influence of materials used to construct nests and the climate zone species where predominately found on species incubation temperatures by evaluating the level of support for four alternative OU models using AIC with a correction for small sample size: (i) ‘nest type’ based on the materials used to construct nests (soil, deadwood, sticks, tree cavities, leaves, workers, or various materials); (ii) ‘climate zone’ where species are predominantly found (subartic, temperate, subtropical or tropical); (iii) the combination of both ‘nest type + climate zone’; and (iv) a phylogenetic ‘null’ model which effectively assumes the evolutionary history of species, and not nesting material or climate zone, had a tangible impact on the optimal incubation temperature exhibited by species. We then applied three phylogenetic OU intercept-only regressions (i.e., with no predictor variable) to make formal inferences on meat ant incubation temperature using data for ‘all species’, ‘temperature species only’ or ‘soil nesting species only’. The intercept estimate computed by these regression models represented the phylogenetic mean estimate and its 95% confidence range for the optimal incubation temperature across species. These estimates provide our second set of inferred optimal incubation temperatures for meat ants. We evaluated the influence of species inclusion on the estimated phylogenetic mean (intercept value) by iteratively removing species from the data set and phylogeny, and recomputing the phylogenetic OU intercept-only regression using the ‘all species’ data set. This implementation was similar to the accuracy index of ancestor state reconstruction to infer an optimal incubation temperature for species not represent in the phylogeny. However, in this context, the computed average of intercept values across the 59 iterative species removals reflects a sensitivity test of the influence individual species might have on the computed phylogenetic mean of optimal incubation temperatures.

#### Inferring changes in nest temperature by depth

Our capacity to embed temperature probes at depths beyond 7 cm was complicated by the need to dig directly into the nest (probes could not be push into the nest beyond the 7 cm length of the metal casing), which would subsequently impact the integrity of the nest architecture, and the aggressiveness of the meat ants themselves (any disturbance to the nest surface induces a hostile swarm of workers; Ord and Blazek 2025). We instead relied on the available published data on nest temperature as a function of depth in species constructing soil nests (Table S2) to extrapolate the likely depth meat ant workers would need to physically relocate broods during peak daytime summer temperatures in order to maintain the brood within the phylogenetically inferred optimal incubation temperature. Formal statistical modelling could not be done with the limited sample size. We instead created a visual model by plotting published nest temperatures as a function of depth and those for the three meat ant nests recorded on the hottest day of the 12-week period.

## Results

### Temperature conditions by nest location

**Fig. 2:**
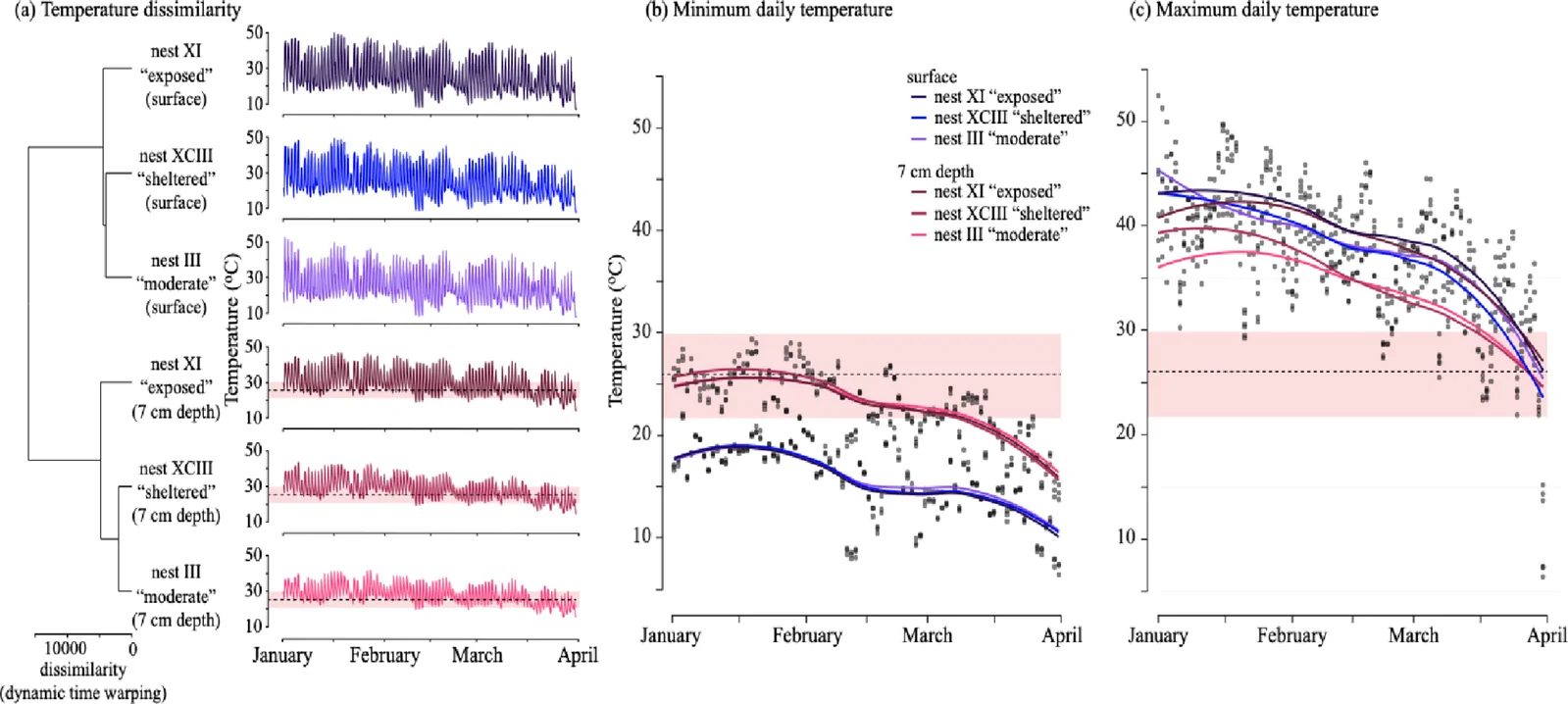
Fluctuations in daytime nest temperature recorded 1 cm above the surface and at a depth of 7 cm during the summer of 2019. Half-hourly data are (**a**) clustered by a dynamic time warping dissimilarity index, which shows two main clusters separating data based on surface versus internal nest temperatures, and a secondary clustering within these that shows nest XI (the ‘exposed’ nest) is the least similar in terms of both surface and internal temperatures compared to the other nests (III ‘moderate’ nest; XCIII ‘sheltered’ nest). Dashed lines on plots of internal nest temperature corresponds to the inferred optimal incubation temperature of 25.7 °C (see Table 3), while the shaded region reflects the credibility range of 21.7–29.8 °C (see Results). Daytime temperatures are summarised as (**b**) minimum and (**c**) maximum daily temperatures with splines taken from the models reported in Table 1

Minimum daily temperatures—typically those recorded overnight near dawn—were consistent among the three nests over the 12-week period (Fig. 2a; Table 1a and S5). This shows all nests cooled overnight to comparable temperatures, with temperatures 1 cm above the nest surface being around 7.2 °C cooler than conditions inside the nest at a depth of 7 cm (Table 1a).

**Table 1:** Differences in (a) minimum and (b) maximum daily temperature recorded 1 cm above the nest surface and at a depth of 7 cm inside the nest for XI (‘exposed’ nest), III (‘moderate’ nest) and XCIII (‘sheltered’ nest) over 12 weeks. The model applied includes a random intercept for the probe recording data (see Table S5 for the equivalent model without a random effect for minimum daily temperature). K refers to the knot number used for the spline smoothing term associated with daily changes in temperature

Variable	Estimate (95% confidence range)	t	F	*p*
*(a) Minimum daily temperature* (°C)				
**Separate**				
Intercept (nest III)	23.22 (22.83, 23.62)	116.21		< 0.0001
Nest XCIII	− 0.01 (− 0.49, 0.47)	− 0.03		0.98
Nest XI	− 0.24 (− 0.72, 0.24)	− 0.98		0.33
Depth (surface vs. internal)	− 7.23 (− 7.63, − 6.84)	− 36.19		< 0.0001
**Overall**				
Nest			0.62	0.54
Depth			1310.01	< 0.0001
Smoothing term (date)			165.5	< 0.0001
*N*_observations, nests, depth_ *=* 540, 3, 2				
k = 5				
*(b) maximum daily temperature* (°C)				
**Separate**				
Intercept (nest III)	34.71 (32.89, 36.53)	37.36		< 0.0001
Nest XCIII	0.09 (− 2.14, 2.32)	0.08		0.93
Nest XI	2.76 (0.53, 4.99)	2.43		0.02
Depth (surface vs. internal)	2.52 (0.70, 4.34)	2.72		0.007
**Overall**				
Nest			3.8	0.02
Depth			7.38	0.007
Smoothing term (date)			131.3	< 0.0001
*N*_observations, nests, depth_ = 540, 3, 2				
k = 5				

However, temperature differences became apparent among nests as the sun warmed nests depending on their location. Nest XI—the most ‘exposed’ nest of the three—had an average maximum daily temperature 2.8 °C hotter than the other two nests across probes at both 1 cm above the surface and 7 cm deep inside the nest (Table 1b). However, inspecting Fig. 2b clarifies this difference to be much larger inside the nest of XI, which was warmer by around 5 °C or more at a depth of 7 cm compared to the other nests. The maximum daily temperature of nest XCIII—the most ‘sheltered’ nest—was not statistically distinguishable from nest III (the ‘moderate’ nest; Table 1b). The average maximum daily temperature at 1 cm above the surface of nests was 2.5 °C hotter than inside the nest at depth of 7 cm (Table 1b), although the ‘exposed’ nest XI routinely recorded internal nest temperatures that converged on surface temperatures (see also ‘Temperature insulation of soil and nest architecture’).

Cluster analysis of half-hourly daily temperatures mirrored the same general trends among nests, with the ‘exposed’ nest XI clustering outside of the ‘sheltered’ nest XCIII and ‘moderate’ nest III, and a clear separation between surface and internal temperatures (Fig. 2a).

### Temperature insulation of soil and nest architecture

**Fig. 3:**
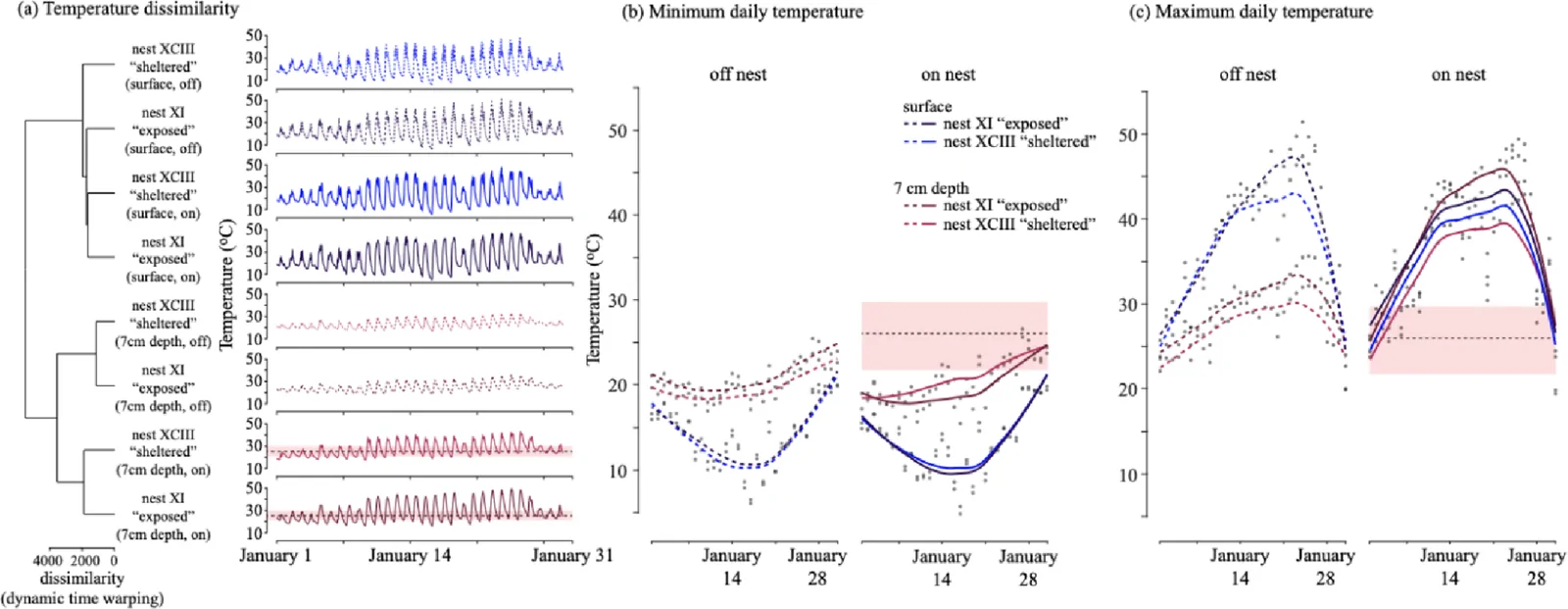
Fluctuations in daytime temperature on and off nests, recorded 1 cm above the nest/soil surface and at a depth of 7 cm during the summer of 2021. Half-hourly data are (**a**) clustered by a dynamic time warping dissimilarity index, which shows two main clusters separating data based on probes positioned just above the nest surface versus inside the nest, and a secondary clustering that separates data based on whether probes were positioned on or off the nest. Dashed lines on plots of internal nest temperature corresponds to the inferred optimal incubation temperature of 26 °C (see Table 3), while the shaded region reflects the credibility range of 21.7–29.8 °C (see Results). Daytime temperatures are summarised as (**b**) minimum and (**c**) maximum daily temperatures with splines taken from the models reported in Table 2

Consistent with temperature recordings in the summer of 2019, there was no statistical evidence for any difference in minimum daily temperatures between nest the ‘exposed’ nest XI and the ‘sheltered’ nest XCIII in the summer of 2021 (Table 2a and S6). Both nests cooled overnight to comparable temperatures (Fig. 3b). For both nests and probe positions on and off the nest over the 4-week period in 2021, the insulating properties of the soil was enough to maintain the minimum daily temperatures on average 6.7 °C warmer at a depth of 7 cm than recorded just above the nest surface (Table 2a; Fig. 3b; see also Table S6). There was also no statistical distinguishable difference in minimum daily temperatures on or off the nest (Table 2a).

**Table 2:** Differences in (a) minimum and (b) maximum daily temperature on and off the nest recorded 1 cm above the nest/soil surface versus at a depth of 7 cm for XI (‘exposed’ nest) and XCIII (‘sheltered’ nest) over 4 weeks. The model applied includes a random intercept for the probe recording data. K refers to the knot number used for the spline smoothing term associated with daily changes in temperature

Variable	Estimate (95% confidence range)	t	*p*
*(a) Minimum daily temperature* (°C)			
Intercept (nest XCIII)	20.54 (19.59, 21.50)	42.16	< 0.0001
Nest XI	0.07 (− 0.89, 1.02)	0.14	0.89
Depth (surface vs. internal)	− 6.67 (− 7.63, − 5.72)	− 13.7	< 0.0001
Location (on vs. off nest)	− 0.43 (1.39, 0.52)	− 0.89	0.38
*N*_observations, nests, depth, location_ = 248, 2, 2, 2			
k = 5			
*(b) Maximum daily temperature* (°C)			
Intercept (nest XCIII)	27.29 (25.86, 28.72)	37.37	< 0.0001
Nest XI	2.46 (1.18, 3.74)	3.76	0.0002
Depth (surface vs. internal)	9.07 (7.26, 10.88)	9.82	< 0.0001
Location (on vs. off nest)	8.03 (6.22, 9.84)	8.69	< 0.0001
Depth * location	− 8.83 (− 11.39, − 6.27)	− 6.76	< 0.0001
*N*_observations, nests, depth, location_ = 248, 2, 2, 2			
k = 5			

As the sun warmed the soil during the day, the maximum daily temperatures recorded for the ‘exposed’ nest XI was 2.5 °C warmer than the ‘sheltered’ nest XCIII (Table 2b), a difference consistent with what was recorded over 12-weeks in the summer of 2019 (see ‘temperature conditions by nest location’ above). Focusing first on the insulating properties of soil off the nest, temperatures at a depth of 7 cm were an average of 9 °C cooler than 1 cm above the soil surface (Table 2b; NB: this cooling effect was as great as almost 15 °C mid-January—Figure 3c), which noticeably reduced the fluctuations in maximum daily temperature in the soil off the nest compared to those being experienced at the surface (Fig. 3a). On nests, however, conditions were quite different. On average, the maximum internal temperature of both nests was only 0.2 °C cooler than just above the nest surface (i.e., the difference of ‘depth’ and ‘depth*location’ parameter estimates in Table 2b: 9.07–8.83 °C). Conditions inside the nest were generally 8.8 °C warmer than at the same depth in the surrounding soil (i.e., the parameter estimate of the ‘depth*location’ parameter term in Table 2b; see also Fig. 3c). This indicates the architecture of the nest resulted in warmer maximum daily temperatures inside the nest than would be expected from the general insulating properties of the soil from which the nest was constructed. Furthermore, Fig. 3c shows only the ‘sheltered’ nest XCIII consistently exhibited internal maximum temperatures cooler than recorded at 1 cm above the nest surface, while the internal temperatures of the ‘exposed’ nest XI routinely peaked at hotter temperatures by several degrees than near the nest surface.

The same trends were identified by cluster analysis of half-hourly daily temperatures (Fig. 3a), with temperatures separating depending on nest ID and where probes were positioned (on- versus off-nest) for surface temperatures, in addition to prominent clustering of temperatures at depth off the nest relative to on the nest. In particular, daily temperature exhibited great fluctuations inside the nest at a 7 cm depth compared to at the same depth in soil off the nest (Fig. 3a).

### Phylogenetic inferences of optimal brood incubation temperature

**Fig. 4:**
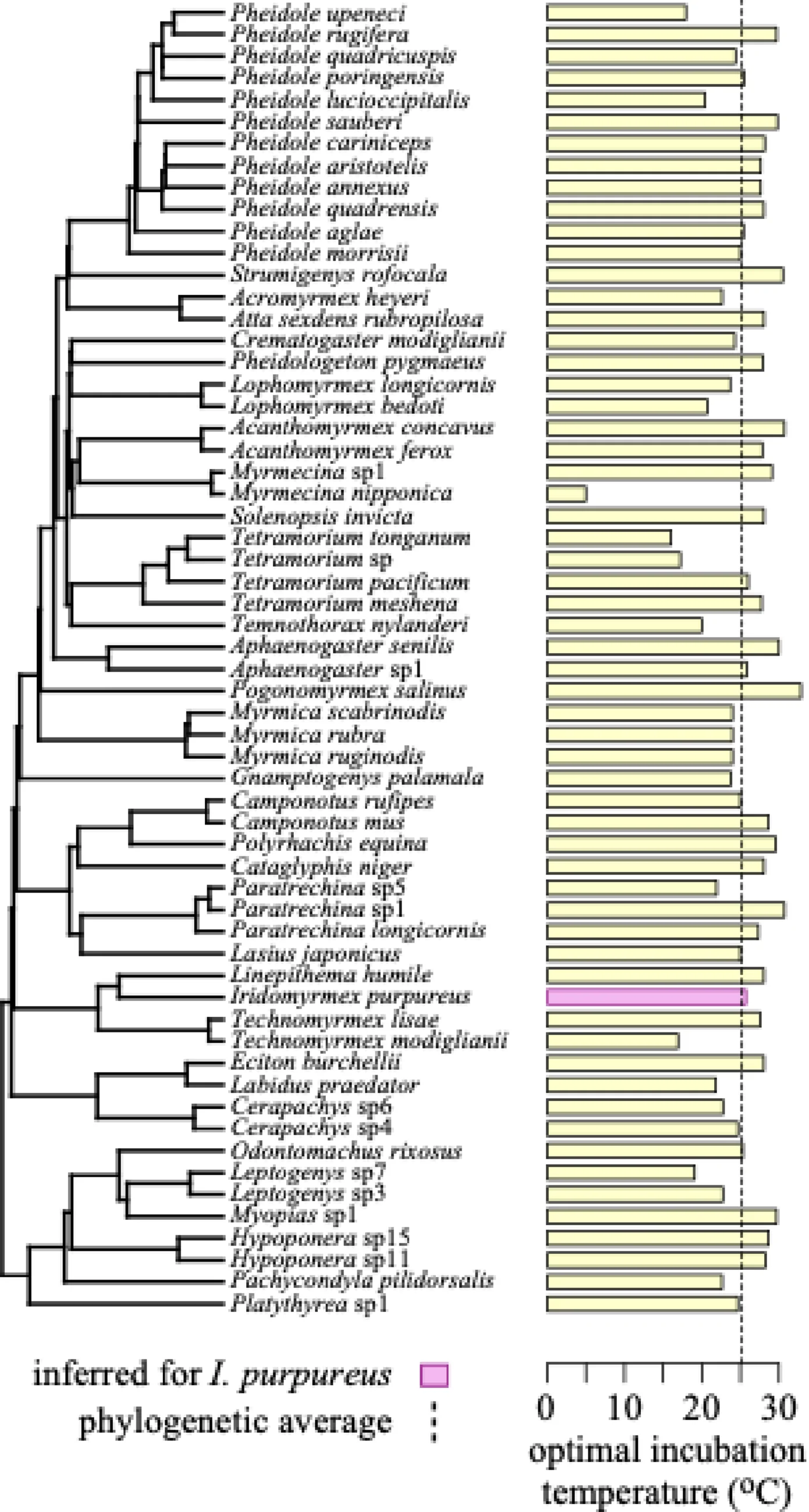
The phylogenetic distribution of brood incubation temperatures reported for ant species in the literature (see Table S1). Data represent either the preferred incubation temperature determined by the position workers move and maintain the brood or the incubation temperature resulting in the maximum rate of development and survival of the brood. Further details are provided in Table S1. Also depicted is the estimate for *I. purpureus* based on a phylogenetic inference taken from a BM evolutionary reconstruction (purple) and the phylogenetic average computed from an OU evolutionary model (dashed line). These estimates are reported in Table 3

Data on the optimal temperature for incubating broods obtained from the literature are shown across species in Fig. 4. The best supported phylogenetic model was the ‘null (intercept only)’ model (Table S7), indicating that neither the material used to construct nests (‘nest type’) or where species were predominantly found (‘climate zone’) accounted for variation in incubation temperature among species. Moreover, inferences of probable brood incubation temperatures for meat ants were broadly consistent irrespective of what dataset were used in analyses or how estimates were inferred (Table 3). For example, the likely optimal incubation temperature for meat ant broods was 25.7 °C when extrapolated from ancestor state reconstructions using a BM evolution model (Table 3a) or 25.2 °C (95% confidence range: 24.0–26.3 °C) based on the phylogenetic mean calculated from an OU evolutionary regression model (Table 3b; see also Fig. 4).

**Table 3:** Phylogenetic estimates of brood incubation temperature based on an inferred (a) estimate for *I. purpureus* taken from a phylogenetic reconstruction using a BM evolutionary model or (b) phylogenetic average computed across all species using an OU evolutionary model. The additional parameter of σ^2^ is a statistical estimate of the likely stochasticity in the evolutionary differentiation of incubation temperature whereas α reflects the strength of selection pushing incubation temperature towards some adaptive optima

Data considered	Predicted temperature (°C)	95% confidence range	σ^2^	α	*N*_species_
*(a) Inferred for I. purpureus from BM model*					
All species	25.7	-	0.6	-	59
Temperate species only	26.1	-	0.4	-	7
Soil nesting species only	26.7	-	0.2	-	13
*(b) Inferred phylogenetic average from OU model*					
All species	25.2	24.0, 26.3	10,542.5	250.5	59
Temperate species only	23.2	17.0, 29.4	55.2	0.4	7
Soil nesting species only	25.1	21.6, 28.9	26.6	0.3	13

Our accuracy index for the ancestor state reconstruction approach suggested, on average, the meat ant inferred optimal incubation temperature was probably within 4.1 °C of its true temperature (based on 59 iterative removals of species with known incubation temperatures obtained from the literature). This provides a credibility range of 21.7 to 29.8 °C around the inferred meat ant incubation temperature of 25.7 °C (i.e., 25.7 ± 4.1 °C). Our sensitivity estimate of the phylogenetic mean to the iterative removal of species from OU evolutionary regressions showed an on average value of 25.18 °C, which was virtually indistinguishable from the phylogenetic mean computed from the all species OU regression model.

We subsequently inferred the likely optimal incubation temperature for meat ant broods to be 25.2–25.7 °C, but also considered the implications of the more extreme inference of the credibility range 21.7–29.8 °C obtained from sensitivity testing of ancestor state reconstructions.

### Tracking optimal incubation temperature by moving broods deeper

In general, summer daily temperatures inside meat nests routinely exceeded the inferred optimal incubation temperature for broods of 25.2–25.7 °C in both 2019 and 2021 (dashed lines in Figs. 2a and 3a). This was the case even if the more extreme upper credibility estimate of 29.8 °C was inferred to be the optimal incubation temperature (shaded area in Figs. 2a and 3a). This suggests meat ant workers would have to physically relocate broods to deeper into the nest to ensure broods were maintained at or near their likely optimal incubation temperature.

**Fig. 5:**
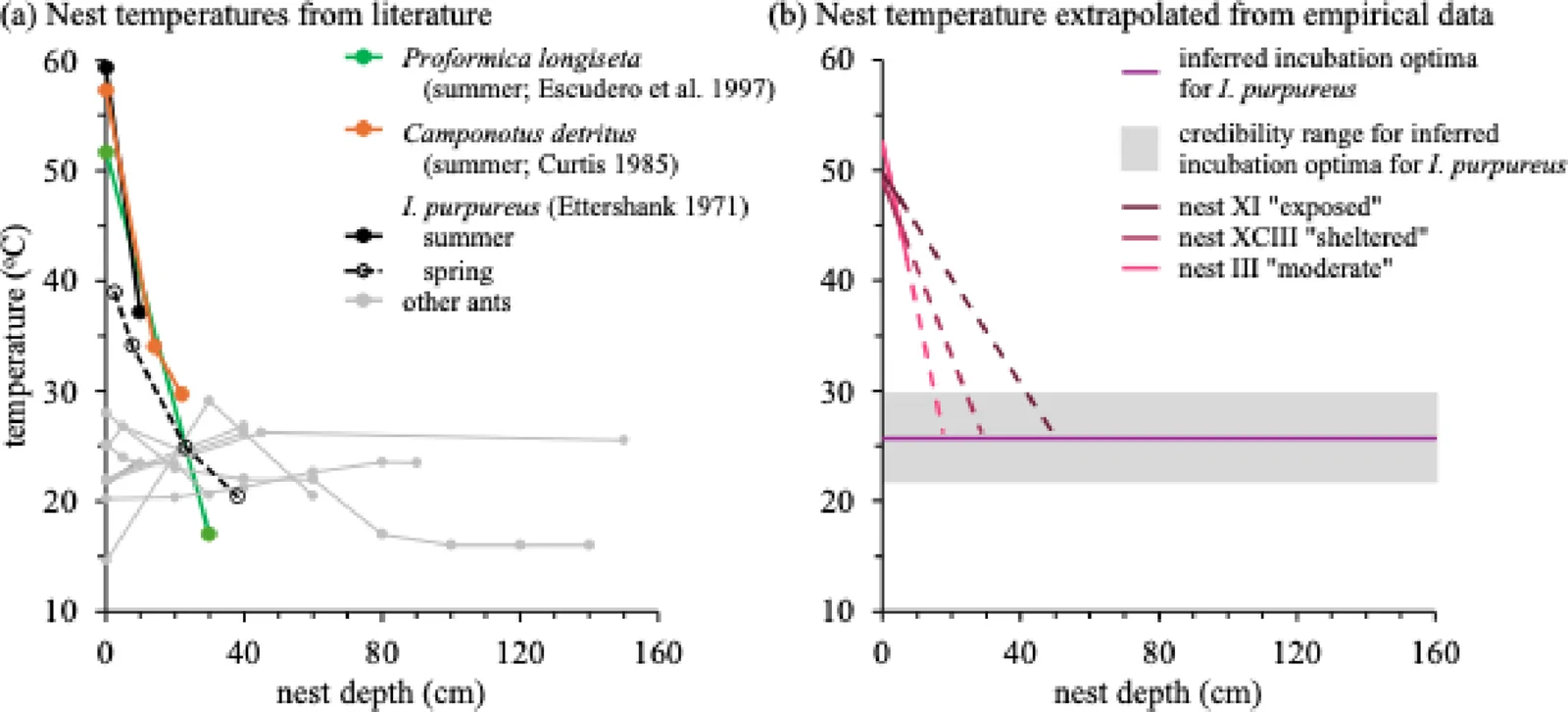
Changes in summertime nest temperature as a function of nest depth obtained from the literature (**a**; see also Table S2), and inferred for meat ants to an optimal incubation temperature of 25.7 °C (**b**; Table 3). Also shown in the right panel (**b**) is the credibility range of 21.7–29.8 °C (shaded region). Mean ant data from the literature (a; *I. purpureus*) includes data for the summertime pilot and springtime primary study reported by Ettershank (1971). Extrapolation for the hottest day recorded in the current study is shown by the coloured dashed lines in right panel (**b**) and indicates the likely depths meat ant workers would have to relocation broods inside the nest in order to maintain the brood at an optimal incubation temperature

Most published reports of internal nest temperatures for soil nest ants were for species typically experiencing comparatively cooler summertime surface temperatures than meat ants (14.5–28.0 °C vs. 49.1–52.5 °C, respectively; Table S2). For these cooler summertime species, there was little consistent change in internal nest temperatures as a function of depth (Fig. 5a). However, two species (*Camponotus detritus* and *Proformica longiseta*; Table S2) were reported to experience similar summertime temperatures to meat ants (51.6–57.2 °C). The single study available for meat ants (Ettershank 1971) measured nest temperatures in both summer and spring, and recorded average maximum daily surface temperatures of 59 °C and 31 °C respectively (Table S2). Nests of the two hot summertime species and from this previous study on meat ants exhibited roughly linear declines in internal temperature with increasing depth, ultimately converging on an inferred optimal incubation temperature (~ 26 °C) at depths of 20–30 cm (or 15–25 cm for the more extreme incubation temperature of ~ 30 °C; Fig. 5a).

Assuming similar linear declines in internal temperature, an extrapolation of summertime meat ant nest maximum daily temperature recorded near the nest surface and internal temperature at 7 cm suggests tangible differences in how deep workers among the three nests would have to relocate broods in order to track the likely optimal incubation temperature of 25.2–25.7 °C. As an example, workers in the ‘exposed’ nest XI would probably have to move broods into chambers nearly 50 cm deep on a hot summer’s day, compared to 20 to 30 cm for workers in the ‘sheltered’ nest XCIII or ‘moderate’ nest III (Fig. 5b). Assuming the more extreme ancestor state credibility range of 21.7–29.8 °C, workers in the ‘exposed’ nest XI would need to move broods anywhere between 42 and 59 cm deep into the nest, compared to 15 to 20 cm for workers in the ‘sheltered’ nest XCIII (Fig. 5b).

## Discussion

Our study targeted three meat ant nests selected from over 100 nests that have been permanently tagged in this population and monitored for over a decade. While this selection of nests offers only a limited sample, and examined only temperature (rather than soil moisture and nest humidity), previous study (Ord and Blazek 2025) showed these three nests should encompass the broad range of conditions experienced by meat ant colonies in this study area. Humidity is known to be important for brood development (e.g., Potts et al. 1984), and rainfall more generally (or lack thereof) seems to be predictive of meat ant colonies budding new nests as a means of attempting to shift the colony to less exposed positions in the environment (Ord 2023; see also Gordon 1992; Sundaram et al. 2022). However, temperature is typically the primary variable considered by studies documenting brood development and survival in ants (see Table S1). Nest location and temperature are also linked to colony survival in meat ants specifically (Ord and Blazek 2025). In this study, we expand on these earlier findings to explore whether nest exposure had tangible impacts on temperature inside the nest and how nest temperature might in turn affect a colony’s capacity to maintain viable broods.

To this end, temperature probes were installed on the three target nests for over two years (Fig. 1). These data showed the thermal properties of the soil from which nests were excavated had the capacity to buffer meat ant colonies from fluctuations in external temperature to an extent. However, neither the insulation of the soil nor the architecture of the nest could compensate for extreme oscillations in external conditions near the nest surface. This was especially problematic for the nest positioned in the most exposed location in the environment (nest XI; Figs. 2 and 3; see also Ord and Blazek 2025). Phylogenetic analysis of brood incubation temperatures across ant species (for which data was available from the literature) inferred a likely optimal temperature range for meat ants centred on 26 °C (Fig. 4; with a credible range of 22–30 °C). In most instances, meat ant workers would probably be able to maintain broods near this optimal incubation temperature if workers physically moved the brood over a range of depths inside the nest (Fig. 5). Nevertheless, the effectiveness of brood relocation would clearly be contingent on the placement of the nest and whether it adequately balanced morning sun exposure and afternoon shade, as well as the availability of brood chambers at varying depths within the nest itself.

Over the course of a typical summer’s day, our data suggests the most exposed nest in our study (nest XI) would have to physically move the brood over a considerably wider depth range (up to 50–60 cm deep) than most other nests in the population (e.g., 15–30 cm deep for XCIII and III). At the least, this would increase the energetic cost associated with brood care and, at worse, reduce the proportion of the brood surviving to adulthood if workers were unable to move the brood deep enough in the nest (Roces and Nunez 1989, 1995; Anderson and Munger 2003; Pranschke and Hooper-Bui 2003; Murdock and Tschinkel 2015; Falibene et al. 2016). We know from excavated meat ant nests (Ettershank 1968; Greenslade 1973; Greaves and Hughes 1974) and photographs of predated nests (e.g., Figure S1) that most galleries are within 15 cm of the nest surface. Evidence from predated nests (e.g., Figure S1) is especially pertinent here given echidnas excavate down to chambers specifically housing eggs and larvae (van Wilgenburg and Elgar 2007a; TJO personal observation). This depth range would not be adequate for maintaining the brood at an incubation optimum on most summer days (e.g., Fig. 2). Nevertheless, some nests have a small portion of galleries up to 30–40 cm deep (Ettershank 1968; Greenslade 1973), which should be suitable for brood relocation during even the hottest summer day recorded in our study. Meat ant galleries are sometimes connected to a long vertical shaft that extends from the upper chambers to a depth of 1–3 m (Ettershank 1968; Greenslade 1973; Greaves and Hughes 1974; NB: few other ant species excavate to such depths; reviewed by O’Fallon et al. 2023). However, these vertical shafts are associated with just one or two chambers (Greenslade 1973). This seems limited for relocating broods deeper into the nest for thermoregulation (these deep chambers are putatively used as winter refuges for the colony; Ettershank 1968).

Further limits to internal brood relocation arise from the general design of meat ant nests. Every hole on the surface of the nest leads to an independent gallery set, with most sets not connected to a deep vertical shaft (Ettershank 1968; Greenslade 1973). This internal modality presumably conveys adaptive benefits through the containment of parasitism (Ettershank 1968). But it also means workers can only move between gallery sets by physically leaving the nest and traveling across the surface to enter through another hole. Meat ant workers have been reported transferring broods across the nest surface in the early morning or evening (Ettershank 1968; see also McIver 1991). Outside of these times, broods are restricted within a given gallery set for the duration of the day. Our internal nest temperature data (Figs. 2 and 3) and field observations of internal nest structure (e.g., Figure S1) imply many meat ant nests would probably have the depth range within a given gallery set to maintain the brood at an optimal (or near optimal) incubation temperature (e.g., ‘moderate’ and ‘sheltered’ nests III and XCIII, respectively; this would likely also be the case for the single meat ant nest surveyed by Ettershank 1971 at a different location and shown in Fig. 5a). But this would not be the case for a nest poorly positioned in the environment or otherwise a nest that becomes exposed because of environmental change (e.g., vegetation dieback from drought; Ord 2023).

In general, though, the range in nest temperatures recorded over summer was lower than temperatures recorded within 1 cm of the surface (Figs. 2 and 3). This manifested as warmer minimum temperatures inside the nest at dawn and (usually) cooler maximum temperatures during the hottest times of the day (Fig. 1; with the exception of the most ‘exposed’ nest XI). Yet nests were still more variable in internal temperature than the surrounding soil at the same depth off the nest (Fig. 3). This was also reported by Ettershank (1971), who recorded a greater range of temperatures inside a single meat ant nest over four days in spring than in the surrounding soil. Although direct comparison of internal nest and off-nest soil temperatures are infrequently made (e.g., in addition to Ettershank (1971), off-nest soil temperatures were only recorded by three of the other eight studies listed in Table S2), our data and those of others (Ettershank 1971; Bristow et al. 1992; Baudier and O’Donnell 2016; Stukalyuk et al. 2020) support a general phenomenon of temperatures inside ant nests not being as tightly modulated as those at the same depth in the surrounding soil. Perhaps this reflects differences in the localised composition of soil used to excavate nests (Greenslade 1973). Yet temperatures inside nests should be expected to be more variable, simply because galleries are connected to entrance holes that are open to conditions at the nest surface. In the case of meat ant nests, entrances are actively maintained open by workers (TJO personal observation). Ambient conditions inside the nest will therefore be more dependent on external conditions than would be expected in the surrounding soil.

In other soil nesting species, aspects of a nest’s architecture can be changed to potentially improve nest thermoregulation depending on the external temperatures experienced by a colony (e.g., O’Fallon et al. 2022; Garcia Ibarra et al. 2024). Meat ants might simply excavate deeper gallery sets to allow the relocation of broods deeper in the nest during summer or to compensate for nest surface exposure. Determining this experimentally (e.g., O’Fallon et al. 2022) would be challenging given the size of meat ant colonies (100,000 to over a million workers per nest; Greaves and Hughes 1974; Ord 2023) and the associated difficulty in maintaining colonies in captivity. In the absence of experimental data, field observation offers some insight on how nest location impacts colony reproduction. First, whole-nest excavations following drought found fewer meat ant nests with larvae in exposed locations (Greaves and Hughes 1974). Cessation of brood production in these instances might have been a plastic response to reduced food availability from drought, but the disproportionate impact on exposed nests implies abiotic conditions within nests were potentially responsible for brood failure. Second, such an impact of nest location on colony reproduction is consistent with the progressive annual decline in size of the most exposed nest in our study (nest XI; number of holes: pre-drought, 2016 = 79; post-drought, 2020 = 55; current, 2026 = 38), whereas other colonies in less exposed locations have maintained their size (e.g., ‘sheltered’ nest XCIII: pre-drought, 2016 = 21; post-drought, 2020 = 35; current, 2026 = 30). Sustained annual declines in nest size typically ends in colony death (Ord 2023; Ord and Blazek 2025). Finally, prolonged environmental change from drought induces colonies to attempt to move nests to less exposed positions through budding (Ord 2023; contrast with a reduction in the establishment of new nests by colonies in other ant species; Couper et al. 2021; Sundaram et al. 2022). Taken together, poor nest placement in meat ants has clear impacts on a colony’s capacity to sustain itself, and the current study implies a key mechanism of this decline could be a colony’s difficulty in properly thermoregulating broods within the nest. Additional, potentially broader factors impacting entire populations of this and other ant species are environmental impacts on the availability of food resources (Ord 2023), especially in the context of drought and ultimately those produced by the climate crisis (see Sundaram et al. 2022).

Soil nesting ant species are considered more resilient to adverse environmental change than arboreal species because of the insulating properties of soil coupled with the capacity to behavioural thermoregulate broods (Parr and Bishop 2022). Our study on an ecologically dominant Australian ant (Anderson and Patel 1994; Gibb and Hochuli 2004) believed to be especially resistant to disturbance (Gibb and Hochuli 2003a; Palfi et al. 2017) shows excavating soil nests comes with its own challenges. Meat ant colonies invest substantially in constructing huge, conspicuous ground nests that are iconic features of the Australian landscape. Colonies can remain in the same nest for decades (Greenslade 1975a; Ord 2023; Ord and Blazek 2025) and the establishment of new nests is rare (Greaves and Hughes 1974; Greenslade 1975b; Ord 2023). While nest budding does occur (Holldobler and Carlin 1985), it seems colonies do so as a means of moving to more favourable locations as an emergency response to severe environmental conditions (extreme and prolonged drought), and with low chances of success (Ord 2023). The placement of nests was previously assumed to depend on maximising the proximity of food resources (Greaves and Hughes 1974; van Wilgenburg and Elgar 2007b), specifically certain eucalypt trees with large concentrations of hemipteran insects from which meat ants harvest honeydew (McIver 1991; Sagata and Gibb 2016). Our data, however, shows nest location is important for maintaining internal nest temperatures as well, with probable flow-on effects for brood incubation. Colonies therefore position a nest in an area that balances solar exposure over the course of a day as a means of nest thermoregulation. This is consistent with the phenomenon of meat ants clustering nests along tree-lines that seem to maximise morning sun-exposure with afternoon shade (Ord 2023), and other ant species that rely on soil temperature to determine excavation sites for soil nests (Bollazzi et al. 2008; Diaz et al. 2013). Unlike arboreally nesting ants or other species that construct nests for short-term occupancy (hours to weeks; Jackson 1957; Smallwood 1982; Gordon 1992; Gibb and Hochuli 2003b; McGlynn et al. 2004; Pinter-Wollman and Brown 2015; Baudier et al. 2019), the longevity of meat ants residing in the same nest increases their vulnerability to prolonged environmental change (Ord 2023; Ord and Blazek 2025). This is in stark contrast to ant species such as the red harvester ant, where decades old colonies are often observed to relocate nests depending on year-to-year fluctuations in vegetation cover affecting nest shading (Sundaram et al. 2022).

The shelter provided by the construction of a refuge is usually a key adaptive benefit for adopting the strategy of a central place forager. Yet when the impacts of environmental change have been considered for central place foragers, it has typically centred on food availability, not the conditions within the refuge itself. We know for example that repeated foraging trips from a fixed location can result in resource halos around refuges (Chase 1998; Weber et al. 2021; Rueffler and Lehmann 2024), and that any environmental factor affecting the distribution of food in the environment will have cascading effects on central place foragers as well (Bakker et al. 2005; Fagan et al. 2007; Patenaude-Monette et al. 2014; Wakefield et al. 2014; Bhattacharyy and Ray 2015; Massardier-Galata et al. 2017; Gicquel et al. 2022; Sundaram et al. 2022). However, our study highlights the placement of the refuge has implications for its adequacy in providing protection from external conditions, especially central place foragers who rely on nests or burrows for reproduction. Refuge locations are finite in most circumstances, either because of competition (Boulay et al. 2010; Sundaram et al. 2022; Ord 2023) or physical constrains on the availability of inhabitable space (Newton 1994; Thomas 2002). Animals within a population might often have to settle for refuges that provide reduced protection or opportunities for reproduction and will subsequently be more vulnerable to environmental change. Our data further suggests this vulnerability is likely to be acerbated for species who invest heavily in refuge construction, because these species are less able to relocate to track even relatively localised changes in conditions (e.g., see Gordon 1992; Sundaram et al. 2022). At a broader scale, the abrupt contraction or geographic shift of habitable zones (e.g., from the climate crisis; Nowrouzi et al. 2018; Couper et al. 2021; see also Boyle et al. 2021 and Gibb et al. 2015, 2019) will not only impact the availability of food resources for a central place forager, but the conditions experienced at the central refuge itself.

## Supplementary Information

Below is the link to the electronic supplementary material.

Supplementary Material 1

## Data Availability

All original data used in analyses are available through the Dryad Digital Repository (Ord and Blazek 2026; https://doi.org/10.5061/dryad.2z34tmq2s). Other data compiled from the literature are presented in Tables S1 and S2.
