# Supplementary material for: Limits on the capacity of an ectotherm to buffer temperature extremes through the construction of a central refuge
Source: Oecologia. 2026 Aug 20;208(9):117. doi: 10.1007/s00442-026-05959-6 (PMC13493446; doi:10.1007/s00442-026-05959-6)
Supplement: Supplementary file 1 [file 442_2026_5959_MOESM1_ESM.docx]

**Electronic Supplemental Material for Ord & Blazek ‘Capacity of an ectotherm to buffer temperature extremes through the construction of a central refuge’**

**ADDITIONAL MATERIALS AND METHODS**

*Phylogenetic positioning of taxa not represented in the Nelsen et al. (2018, 2023) phylogeny*

We used several methods to phylogenetically position unrepresented taxa. First, taxa replaced species of the same genus with the closest geographic distribution (based on data from www.AntiWiki.org). Second, instances where there was only a single species of the same genus that was also included in our data set, taxa were positioned immediately adjacent to this species with a branch length averaged from those of the closest phylogenetic genus. This average included only branches within this genus that lead directly to terminal taxa at the tip of the phylogeny (i.e., internal branch lengths within the genus were not included in the calculation). Third, taxa replaced the single species of the same genus represented on the phylogeny if this species was not included in our data set. Fourth, if geographic information was not available for the unrepresented taxa or species represented on the phylogeny, taxa arbitrarily replaced a species within the same genus on the phylogeny. Finally, in one instance, a taxa (*Solenopsis invicta*) replaced a species (*Mayriella transfuga*) represented on the Nelsen phylogeny based on the phylogenetic information reported in Moreau et al (2006). Details on the phylogenetic assignment of individual taxa are describe below.

*Aphaenogaster* sp. 1 nests used to estimate brood incubation temperature were originally collected by Mezger and Pfeiffer (2010) in Sarawak, Malaysia and the species was subsequently positioned on the phylogeny in replace of *Aphaenogaster pythia*, the closest geographic species of the same genus.

*Atta sexdens rubropilosa* nests used to estimate brood incubation temperature were collected in Sao Paulo State, Brazil by Camargo et al. (2016) and the species was positioned on the phylogeny in replace of *Atta cephalotes*, the closest geographic species of the same genus.

*Acanthomyrmex concavus* was positioned on the phylogeny as a sister species to *A. ferox*, with a branch length averaged from those of the phylogenetically adjacent genus *Lordomyrma*.

*Camponotus mus* nests used to estimate brood incubation temperature were collected in Uruguay by Falibene et al. (2016; see also Roces and Nunez 1995) and the species was positioned on the phylogeny in replace of *C. renggeri,* the closest geographic species of the same genus.

*Cataglyphis niger* replaced *C. aenescens*, the single species from the genus represented on the phylogeny.

*Cerapachys* sp. 4 replaced *C. jacobsoni*, the single species from the genus represented on the phylogeny.

*Cerapachys* sp. 6 was positioned on the phylogeny as a sister species to *Cerapachys* sp. 4 (see above), with a branch length averaged from those of the phylogenetically adjacent genus *Aenictus*.

*Gnamptogenys palamala* replaced *G. striatula*, the single species from the genus represented on the phylogeny.

*Hypoponera* sp. 11 arbitrarily replaced *H. johannae* because there was no geographic information available for species represented on the phylogeny from the same genus.

*Hypoponera* sp. 15 was positioned on the phylogeny as a sister species to *Hypoponera* sp. 11 (see above), with a branch length averaged of those species from the same genus represented on the phylogeny.

*Lasius japonicus* arbitrarily replaced *L. latipes* because there was no geographic information available for species represented on the phylogeny from the same genus.

*Leptogenys* sp. 3 nests used to estimate brood incubation temperature were originally collected by Mezger and Pfeiffer (2010) in Sarawak, Malaysia and the species was subsequently positioned on the phylogeny in replace of *L. iridescens*, the closest geographic species of the same genus.

*Leptogenys* sp. 7 nests used to estimate brood incubation temperature were originally collected by Mezger and Pfeiffer (2010) in Sarawak, Malaysia and the species was subsequently positioned on the phylogeny in replace of *L. crassicornis*, the closest geographic species of the same genus.

*Linepithema humile* arbitrarily replaced *L. angulatum* because there was no geographic information available for species represented on the phylogeny from the same genus.

*Lophomyrmex bedoti* replaced *L. ambiguus*, the single species from the genus represented on the phylogeny.

*Lophomyrmex longicornis* was positioned on the phylogeny as a sister species to *L. bedoti* (see above), with a branch length averaged of those species from the same genus represented on the phylogeny.

*Myopias* sp. 1 replaced *Myopias tenuis*, the single species from the genus represented on the phylogeny.

*Myrmecina nipponica* arbitrarily replaced *M. americana* because there was no geographic information available for species represented on the phylogeny from the same genus.

*Myrmecina* sp. 1 arbitrarily replaced *M. graminicola* because there was no geographic information available for species represented on the phylogeny from the same genus.

*Myrmica rubra* nests used to estimate brood incubation temperature were originally collected by Kipyatkov and Lopatina (2002) in Ukraine and the species was subsequently positioned on the phylogeny in replace of *M. punctiventris* and immediately adjacent to *M. ruginodis*, both of which are the closest geographic species of the same genus.

*Pachycondyla pilidorsalis* replaced *P. harpax*, the single species from the genus represented on the phylogeny.

*Paratrechina longicornis* arbitrarily replaced *P. zanjensis* because there was no geographic information available for species represented on the phylogeny from the same genus.

*Paratrechina* sp. 1 arbitrarily replaced *P. antsingy* because there was no geographic information available for species represented on the phylogeny from the same genus.

*Paratrechina* sp. 5 arbitrarily positioned as the immediate sister taxa to *Paratrechina* sp. 1, with a branch length averaged from that of *Paratrechina longicornis* and *Paratrechina* sp. 1 (see above).

The following species where brood incubation temperature were estimated from nests originally collected by Mezger and Pfeiffer (2010) in Sarawak, Malaysia, arbitrarily replaced species within the same genus that similarly had geographic distributions within Sarawak (e.g., *Pheidole lucioccipitalis*, *P. morrisi* and *P. quadrensis*): *P. aglae* replaced *P. militicida*; *P. annexus* replaced *P. sabahna*; *P. aristotelis* replaced *P. nodus*; *P. cariniceps* replaced *P. dugasi*; *P. poringensis* replaced *P. tjibodana*; *P. quadricuspis* replaced *P. jacobsoni*; *P. rugifera* replaced *P. dea*; and *P. upenec* replaced *P. nigeriensis.*

*Pheidologeton pygmaeus* is a synonym of *Carebara pygmaea* and arbitrarily replaced *C. vidua* because there was no geographic information available for species represented on the phylogeny from the same genus.

*Platythyrea* sp. 1 arbitrarily replaced *P. conradti* because there was no geographic information available for species represented on the phylogeny from the same genus.

*Pogonomyrmex salinus* arbitrarily replaced *P. saucius* because there was no geographic information available for species represented on the phylogeny from the same genus.

*Strumigenys rofocala* arbitrarily replaced *S. exiguaevitae* because there was no geographic information available for species represented on the phylogeny from the same genus.

*Technomyrmex lisae* arbitrarily replaced *T. innocens* because there was no geographic information available for species represented on the phylogeny from the same genus.

*Technomyrmex modiglianii* arbitrarily replaced *T. anterops* because there was no geographic information available for species represented on the phylogeny from the same genus.

*Temnothorax nylanderi* arbitrarily replaced *T. kutteri* because there was no geographic information available for species represented on the phylogeny from the same genus.

The following species arbitrarily replaced a group of closely-related species within the same genus because there was no geographic information available for species represented on the phylogeny from the same genus: *Tetramorium meshena* replaced *T. scytalum*; *T. pacificum* replaced *T. taylori*; *Tetramorium* sp (*near vertigum*) replaced *T. venator*; and *T. tonganum* replaced *T. validiusculum*.

*Solenopsis invicta* replaced *Mayriella transfuga* because this species was identified as a sister taxa to this latter species by Moreau et al (2006).

SUPPLMENTARY REFERENCES

Moreau, C. S., C. D. Bell, R. Vila, S. B. Archibald, and N. E. Pierce. 2006. Phylogeny of the ants: diversification in the age of angiosperms. Science **312**:101–104.

Nelsen, M. P., R. H. Ree, and C. S. Moreau. 2018. Ant–plant interactions evolved through increasing interdependence. Proceedings of the National Academy of Sciences **115**:12253–12258.

Nelsen, M. P., C. S. Moreau, C. Kevin Boyce, and R. H. Ree. 2023. Macroecological diversification of ants is linked to angiosperm evolution. Evol Lett **7**:79–87.

**Table S1.** Brood incubation temperatures reported in the literature for 59 ant taxa. Information on region and nest type were used in evolutionary regressions as additional predictor variables. Details on how authors determined the ideal (optimal) incubation temperature for a species is clarified in the ‘notes’ column.

| species | location | region | nest type | optimal incubation temperature (oC) | setting | notes | source |
| --- | --- | --- | --- | --- | --- | --- | --- |
| *Acanthomyrmex concavus* | Sarawak, Malaysia | tropical | tree | 30.7 | lab | preferred temperature for brood | Mezger and Pfeiffer 2010 |
| *Acanthomyrmex ferox* | Sarawak, Malaysia | tropical | various | 27.8 | lab | preferred temperature for brood | Mezger and Pfeiffer 2010 |
| *Acromyrmex heyeri* | Canelones, Uruguay | temperate | various | 22.6 | lab | preferred temperature for brood, taken as the midpoint between two temperature preferences (20.8C and 24.4C) estimated based on 10C and 37C starting points | Bollazzi and Roces 2002 |
| *Aphaenogaster senilis* | Iberian Peninsula | temperate | soil | 30 | lab | optimal temperature for shortest number of days to produce workers based on three treatments (24C, 27C and 30C) | Boulay et al. 2009 |
| *Aphaenogaster* sp. 1 | Sarawak, Malaysia | tropical | deadwood | 25.8 | lab | preferred temperature for brood | Mezger and Pfeiffer 2010 |
| *Atta sexdens rubropilosa* | Sao Paulo State, Brazil | tropical | soil | 28 | lab | optimal temperature for worker production based on four temperature treatments (15C, 20C, 24C and 28C) | Camargo et al. 2016 |

**Table S1.** Continued.

| species | location | region | nest type | optimal incubation temperature (oC) | setting | notes | source |
| --- | --- | --- | --- | --- | --- | --- | --- |
| *Camponotus mus* | La Coronilla, Uruguay / Buenos Aires, Argentina | subtropical | various | 28.6 | lab | Falibene: optimal temperature for colonies that produce pupae that survive; Roces and Nunez: preferred temperature for brood, taken as the midpoint between two means (27.5C and 30.8C)) used Falibene because of larger sample size | Falibene et al. 2016; Roces and Nunez 1995 |
| *Camponotus rufpes* | Sao Paulo State, Brazil | tropical | deadwood | 25 | lab | preferred temperature for brood | Roces and Nunez 1995 |
| *Cataglyphis niger* | Tel Aviv, Israel | subtropical | soil | 28 | lab | optimal temperature based on total duration starting at 28C then dropped to 22C | Bar et al. 2022 |
| *Cerapachys* sp. 4 | Sarawak, Malaysia | tropical | various | 24.7 | lab | preferred temperature for brood | Mezger and Pfeiffer 2010 |
| *Cerapachys* sp. 6 | Sarawak, Malaysia | tropical | various | 22.7 | lab | preferred temperature for brood | Mezger and Pfeiffer 2010 |
| *Crematogaster modiglianii* | Sarawak, Malaysia | tropical | deadwood | 24.3 | lab | preferred temperature for brood | Mezger and Pfeiffer 2010 |
| *Eciton burchellii* | Barro Colorado, Panama Canal | tropical | none | 28 | field | preferred temperature for brood based on location of the majority of the brood in natural nests | Jackson 1957; Franks 1989 |

**Table S1.** Continued.

| species | location | region | nest type | optimal incubation temperature (oC) | setting | notes | source |
| --- | --- | --- | --- | --- | --- | --- | --- |
| *Gnamptogenys palamala* | Sarawak, Malaysia | tropical | deadwood | 23.8 | lab | preferred temperature for brood | Mezger and Pfeiffer 2010 |
| *Hypoponera* sp. 11 | Sarawak, Malaysia | tropical | leaves | 28.2 | lab | preferred temperature for brood | Mezger and Pfeiffer 2010 |
| *Hypoponera* sp. 15 | Sarawak, Malaysia | tropical | deadwood | 28.6 | lab | preferred temperature for brood | Mezger and Pfeiffer 2010 |
| *Labidus praedator* | Costa Rica | tropical | soil | 21.8 | field | preferred temperature for brood based on location of the majority of the brood in natural nests at 30–40 cm depth | Baudier and O'Donnell 2016 |
| *Lasius japonicus* | Okayama, Japan | subtropical | various | 25 | lab | optimal rearing temperature producing larvae or emerged pupae based on three to four temperatures used (15, 17.5, 20 and 25) | Nakamura et al. 2017; Kuroki et al. 2018; Kuroki et al. 2019 |
| *Leptogenys* sp. 3 | Sarawak, Malaysia | tropical | various | 22.7 | lab | preferred temperature for brood | Mezger and Pfeiffer 2010 |
| *Leptogenys* sp. 7 | Sarawak, Malaysia | tropical | leaves | 19.1 | lab | preferred temperature for brood | Mezger and Pfeiffer 2010 |
| *Linepithema humile* | Iberian Peninsula | temperate | various | 28 | lab | optimal temperature for oviposition | Abril et al. 2008 |
| *Lophomyrmex bedoti* | Sarawak, Malaysia | tropical | various | 20.7 | lab | preferred temperature for brood | Mezger and Pfeiffer 2010 |

**Table S1.** Continued.

| species | location | region | nest type | optimal incubation temperature (oC) | setting | notes | source |
| --- | --- | --- | --- | --- | --- | --- | --- |
| *Lophomyrmex longicornis* | Sarawak, Malaysia | tropical | various | 23.7 | lab | preferred temperature for brood | Mezger and Pfeiffer 2010 |
| *Myopias* sp. 1 | Sarawak, Malaysia | tropical | deadwood | 29.7 | lab | preferred temperature for brood | Mezger and Pfeiffer 2010 |
| *Myrmecina nipponica* | Hokkaido University, Japan | temperate | soil | 5 | lab | optimal temperature for worker and queen production based on two temperature treatments (5 and 20C) | Murakami et al. 2002 |
| *Myrmecina* sp. 1 | Sarawak, Malaysia | tropical | tree | 29.1 | lab | preferred temperature for brood | Mezger and Pfeiffer 2010 |
| *Myrmica rubra* | Ukraine / Russia | temperate | deadwood | 24 | lab | optimal temperature for production of brood, both in number and shortest development time, based on six treatment temperatures (16, 18, 20, 22, 24 and 26C) | Kipyatkov and Lopatina 2002 |
| *Myrmica ruginodis* | Ukraine / Russia | subartic | deadwood | 24 | lab | optimal temperature for production of brood, both in number and shortest development time, based on six treatment temperatures (16, 18, 20, 22, 24 and 26C) | Kipyatkov and Lopatina 2002 |

**Table S1.** Continued.

| species | location | region | nest type | optimal incubation temperature (oC) | setting | notes | source |
| --- | --- | --- | --- | --- | --- | --- | --- |
| *Myrmica scabrinodis* | Russia | subartic | soil | 24 | lab | optimal temperature for production of brood, both in number and shortest development time, based on six treatment temperatures (16, 18, 20, 22, 24 and 26C) | Kipyatkov and Lopatina 2002 |
| *Odontomachus rixosus* | Sarawak, Malaysia | tropical | deadwood | 25.3 | lab | preferred temperature for brood | Mezger and Pfeiffer 2010 |
| *Pachycondyla pilidorsalis* | Sarawak, Malaysia | tropical | various | 22.6 | lab | preferred temperature for brood | Mezger and Pfeiffer 2010 |
| *Paratrechina longicornis* | Sarawak, Malaysia | tropical | various | 27.3 | lab | preferred temperature for brood | Mezger and Pfeiffer 2010 |
| *Paratrechina* sp. 1 | Sarawak, Malaysia | tropical | various | 30.7 | lab | preferred temperature for brood | Mezger and Pfeiffer 2010 |
| *Paratrechina* sp. 5 | Sarawak, Malaysia | tropical | deadwood | 21.9 | lab | preferred temperature for brood | Mezger and Pfeiffer 2010 |
| *Pheidole aglae* | Sarawak, Malaysia | tropical | various | 25.4 | lab | preferred temperature for brood | Mezger and Pfeiffer 2010 |
| *Pheidole annexus* | Sarawak, Malaysia | tropical | various | 27.6 | lab | preferred temperature for brood | Mezger and Pfeiffer 2010 |
| *Pheidole aristotelis* | Sarawak, Malaysia | tropical | various | 27.6 | lab | preferred temperature for brood | Mezger and Pfeiffer 2010 |
| *Pheidole cariniceps* | Sarawak, Malaysia | tropical | various | 28.1 | lab | preferred temperature for brood | Mezger and Pfeiffer 2010 |

**Table S1.** Continued.

| species | location | region | nest type | optimal incubation temperature (oC) | setting | notes | source |
| --- | --- | --- | --- | --- | --- | --- | --- |
| *Pheidole lucioccipitalis* | Sarawak, Malaysia | tropical | various | 20.4 | lab | preferred temperature for brood | Mezger and Pfeiffer 2010 |
| *Pheidole morrisi* | Florida, USA | subtropical | soil | 25 | field | preferred temperature for brood, based on exervated nests with corresponding internal temperatures at depth where brood was primarily found | Murdock and Tschinkel 2015 |
| *Pheidole poringensis* | Sarawak, Malaysia | tropical | sticks | 25.5 | lab | preferred temperature for brood | Mezger and Pfeiffer 2010 |
| *Pheidole quadrensis* | Sarawak, Malaysia | tropical | various | 28 | lab | preferred temperature for brood | Mezger and Pfeiffer 2010 |
| *Pheidole quadricuspis* | Sarawak, Malaysia | tropical | deadwood | 24.4 | lab | preferred temperature for brood | Mezger and Pfeiffer 2010 |
| *Pheidole rugifera* | Sarawak, Malaysia | tropical | sticks | 29.7 | lab | preferred temperature for brood | Mezger and Pfeiffer 2010 |
| *Pheidole sauberi* | Sarawak, Malaysia | tropical | leaves | 29.8 | lab | preferred temperature for brood | Mezger and Pfeiffer 2010 |
| *Pheidole upeneci* | Sarawak, Malaysia | tropical | sticks | 17.9 | lab | preferred temperature for brood | Mezger and Pfeiffer 2010 |
| *Pheidologeton pygmaeus* | Sarawak, Malaysia | tropical | deadwood | 27.9 | lab | preferred temperature for brood | Mezger and Pfeiffer 2010 |
| *Platythyrea* sp. 1 | Sarawak, Malaysia | tropical | various | 24.7 | lab | preferred temperature for brood | Mezger and Pfeiffer 2010 |

**Table S1.** Continued.

| species | location | region | nest type | optimal incubation temperature (oC) | setting | notes | source |
| --- | --- | --- | --- | --- | --- | --- | --- |
| *Pogonomyrmex salinus* | Idaho, USA | temperate | soil | 32.8 | lab | preferred temperature for brood, taken as the midpoint between two temperature depths where most of the brood was found (29.2, 36.4) | Anderson and Munger 2003 |
| *Polyrhachis (Myrma) equina* | Sarawak, Malaysia | tropical | deadwood | 29.6 | lab | preferred temperature for brood | Mezger and Pfeiffer 2010 |
| *Solenopsis invicta* | Louisiana, USA | subtropical | soil | 28 | field | presence of broods in nests in the field (preferred over Porter 1988 who tested a small number of colonies in the lab) | Pranschke and Hooper-Bui 2003; Porter 1988 |
| *Strumigenys rofocala* | Sarawak, Malaysia | tropical | tree | 30.5 | lab | preferred temperature for brood | Mezger and Pfeiffer 2010 |
| *Technomyrmex lisae* | Sarawak, Malaysia | tropical | various | 27.5 | lab | preferred temperature for brood | Mezger and Pfeiffer 2010 |
| *Technomyrmex modiglianii* | Sarawak, Malaysia | tropical | various | 16.9 | lab | preferred temperature for brood | Mezger and Pfeiffer 2010 |
| *Temnothorax nylanderi* | Paris, France | temperate | various | 20 | lab | optimal temperature for worker production based on two temperature treatments (20, 27) | Molet et al. 2017 |
| *Tetramorium meshena* | Sarawak, Malaysia | tropical | deadwood | 27.7 | lab | preferred temperature for brood | Mezger and Pfeiffer 2010 |

**Table S1.** Continued.

| species | location | region | nest type | optimal incubation temperature (oC) | setting | notes | source |
| --- | --- | --- | --- | --- | --- | --- | --- |
| *Tetramorium pacificum* | Sarawak, Malaysia | tropical | deadwood | 26 | lab | preferred temperature for brood | Mezger and Pfeiffer 2010 |
| *Tetramorium* sp (*vertigum*) | Sarawak, Malaysia | tropical | deadwood | 17.2 | lab | preferred temperature for brood | Mezger and Pfeiffer 2010 |
| *Tetramorium tonganum* | Sarawak, Malaysia | tropical | sticks | 16 | lab | preferred temperature for brood | Mezger and Pfeiffer 2010 |

TABLE S1 REFERENCES

Abril, S., J. Oliveras, and C. Gomez. 2008. Effect of temperature on the oviposition rate of Argentine ant queens (Linepithema humile Mayr) under monogynous and polygynous experimental conditions. J Insect Physiol **54**:265–272.

Anderson, K. E., and J. C. Munger. 2003. Effect of temperature on brood relocation in *Pogonomyrmex salinus* (Hymenoptera: Formicidae). Western North American Naturalist **63**:122–128.

Bar, A., L. Shalev, and I. Scharf. 2022. Egg-Laying Behavior of Cataglyphis niger Ants Is Influenced More Strongly by Temperature Than Daylength. Biology (Basel) **11**.

Baudier, K. M., and S. O’Donnell. 2016. Structure and thermal biology of subterranean army ant bivouacs in tropical montane forests. Insectes Sociaux **63**:467–476.

Bollazzi, M., and F. Roces. 2002. Thermal preference for fungus culturing and brood location by workers of the thatching grass-cutting ant *Acromyrmex heyeri*. Insectes Sociaux **49**:153–157.

Boulay, R., X. I. M. CerdÁ, A. Fertin, K. Ichinose, and A. Lenoir. 2009. Brood development into sexual females depends on the presence of a queen but not on temperature in an ant dispersing by colony fission, Aphaenogaster senilis. Ecological Entomology **34**:595–602.

Camargo, R., L. C. Forti, C. A. O. Matos, N. Caldato, and O. S. Fonseca. 2016. Is the initial nest depth adapted to favorable conditions for the incipient colony in leaf-cutting ants? Sociobiology **63**.

Falibene, A., F. Roces, W. Rossler, and C. Groh. 2016. Daily Thermal Fluctuations Experienced by Pupae via Rhythmic Nursing Behavior Increase Numbers of Mushroom Body Microglomeruli in the Adult Ant Brain. Front Behav Neurosci **10**:73.

Franks, N. R. 1989. Thermoregulation in army ant bivouacs. Physiological Entomology **14**:397–404.

Jackson, W. B. 1957. Microclimatic patterns in the army ant bivouac. Ecology **38**:276–285.

Kipyatkov, V. E., and E. B. Lopatina. 2002. Reaction norm in response to temperature may change to adapt rapid brood development to boreal and subarctic climates in Myrmica ants (Hymenoptera: Formicidae). European Journal of Entomology **99**:197–208.

Kuroki, I., J. Tagawa, and K. Nakamura. 2018. Endogenous periodicity in egg-number fluctuation in Lasius japonicus (Formicidae). Biological Rhythm Research:1–11.

Kuroki, I., J. Tagawa, and K. Nakamura. 2019. Geographic variation of temperature effects on initial colony development of Lasius japonicus (Hymenoptera: Formicidae). Applied Entomology and Zoology **54**:175–183.

Mezger, D., and M. Pfeiffer. 2010. Is nest temperature an important factor for niche partitioning by leaf-litter ants (Hymenoptera: Formicidae) in Bornean rain forests? Journal of Tropical Ecology **26**:445–455.

Molet, M., R. Peronnet, S. Couette, C. Canovas, and C. Doums. 2017. Effect of temperature and social environment on worker size in the ant Temnothorax nylanderi. J Therm Biol **67**:22–29.

Murakami, T., K. Ohkawara, and S. Higashi. 2002. Morphology and developmental plasticity of reproductive females in *Myrmecina nipponica* (Hymenoptera: Formicidae). Annals of the Entomological Society of America **95**:577–582.

Murdock, T. C., and W. R. Tschinkel. 2015. The life history and seasonal cycle of the ant, Pheidole morrisi Forel, as revealed by wax casting. Insectes Sociaux **62**:265–280.

Nakamura, K., M. Fujiyama, and K. Ohta. 2016. Effect of temperature on queen oviposition and seasonal colony development in Lasius japonicus (Hymenoptera: Formicidae). Applied Entomology and Zoology **52**:107–112.

Porter, S. D. 1988. Impact of temperature on colony growth and developmental rates of the ant, *Solenopsis invicta*. Journal of Insect Physiology **34**:1127–1133.

Pranschke, A. M., and L. M. Hooper-Bui. 2003. Influence of abiotic factors on red imported fire ant (Hymenoptera: Formicidae) mound population ratings in Louisiana. Environmental Entomology **32**:204–207.

Roces, F., and J. A. Nunez. 1995. Thermal sensitivity during brood care in workers of two *Camponotus* ant species: circadian variation and its ecological correlates. Journal of Insect Physiology **41**:659–669.

**Table S2.** Nest temperature as a function of depth across ant taxa that excavate soil nests. Data were compiled from reports measuring nest temperatures during summer. In the case of the meat ant (*I. purpureus*), data were extracted for the main survey conducted in spring (September, over 4 days) as well as the pilot survey conducted in summer (February, over 2 days; Ettershank 1971).

| species | depth (cm) | temperature estimate | nest temperature (^o^C) | N_nest_ | source |
| --- | --- | --- | --- | --- | --- |
| *Atta sexdens rubropilosa* | 0 | mean, daily | 24.91 | 1 | Camargo et al. (2016) |
| *Atta sexdens rubropilosa* | 5 | mean, daily | 26.77 | 1 | Camargo et al. (2016) |
| *Atta sexdens rubropilosa* | 25 | mean, daily | 24.21 | 1 | Camargo et al. (2016) |
| *Atta sexdens rubropilosa* | 45 | mean, daily | 26.22 | 1 | Camargo et al. (2016) |
| *Atta sexdens rubropilosa* | 150 | mean, daily | 25.53 | 1 | Camargo et al. (2016) |
| *Camponotus detritus* | 0 | point sample | 57.25 | 1 | Curtis (1985) |
| *Camponotus detritus* | 14 | point sample | 34.01 | 1 | Curtis (1985) |
| *Camponotus detritus* | 22 | point sample | 29.7 | 1 | Curtis (1985) |
| *Formica exsectoides* | 0 | point sample | 28 | 1 | Bristow et al (1992) |
| *Formica exsectoides* | 20 | point sample | 23 | 1 | Bristow et al (1992) |
| *Formica exsectoides* | 40 | point sample | 22 | 1 | Bristow et al (1992) |
| *Formica exsectoides* | 60 | point sample | 22 | 1 | Bristow et al (1992) |
| *Formica exsectoides* | 80 | point sample | 17 | 1 | Bristow et al (1992) |
| *Formica exsectoides* | 100 | point sample | 16 | 1 | Bristow et al (1992) |
| *Formica exsectoides* | 120 | point sample | 16 | 1 | Bristow et al (1992) |
| *Formica exsectoides* | 140 | point sample | 16 | 1 | Bristow et al (1992) |
| *Formica polyctena* | 0 | mean, daily | 25.155 | 1 | Brandt (1980) |
| *Formica polyctena* | 5 | mean, daily | 23.92 | 1 | Brandt (1980) |
| *Formica polyctena* | 10 | mean, daily | 23.457 | 1 | Brandt (1980) |
| *Formica polyctena* | 20 | mean, daily | 23.303 | 1 | Brandt (1980) |
| *Formica polyctena* | 30 | mean, daily | 20.53 | 1 | Brandt (1980) |
| *Formica rufa* | 0 | mean, daily | 14.55 | 1 | Rosengren et al. (1987) |
| *Formica rufa* | 30 | mean, daily | 29.101 | 1 | Rosengren et al. (1987) |
| *Formica rufa* | 60 | mean, daily | 20.506 | 1 | Rosengren et al. (1987) |
| *Formica rufa* | 0 | mean | 21.6 | 5 | Stukalyuk et al. (2020) |
| *Formica rufa* | 10 | mean | 23.7 | 5 | Stukalyuk et al. (2020) |
| *Formica rufa* | 0 | mean | 21.8 | 5 | Stukalyuk et al. (2020) |
| *Formica rufa* | 40 | mean | 26.2 | 5 | Stukalyuk et al. (2020) |
| *Formica rufa* | 0 | mean | 22 | 5 | Stukalyuk et al. (2020) |
| *Formica rufa* | 40 | mean | 26.8 | 5 | Stukalyuk et al. (2020) |

**Table S2.** Continued.

| species | depth (cm) | temperature estimate | nest temperature (^o^C) | N_nest_ | source |
| --- | --- | --- | --- | --- | --- |
| *Formica rufa* | 0 | median | 20.206 | 10 | Balzani et al. (2022) |
| *Formica rufa* | 20 | median | 20.348 | 10 | Balzani et al. (2022) |
| *Formica rufa* | 40 | median | 21.234 | 10 | Balzani et al. (2022) |
| *Formica rufa* | 60 | median | 22.658 | 10 | Balzani et al. (2022) |
| *Formica rufa* | 80 | median | 23.544 | 10 | Balzani et al. (2022) |
| *Formica rufa* | 90 | median | 23.465 | 10 | Balzani et al. (2022) |
| *Iridomyrmex purpureus* | 2.5 | mean, daily max (spring) | 38.95 | 1 | Ettershank (1971) |
| *Iridomyrmex purpureus* | 7.6 | mean, daily max (spring) | 34.125 | 1 | Ettershank (1971) |
| *Iridomyrmex purpureus* | 23 | mean, daily max (spring) | 24.9 | 1 | Ettershank (1971) |
| *Iridomyrmex purpureus* | 38 | mean, daily max (spring) | 20.425 | 1 | Ettershank (1971) |
| *Iridomyrmex purpureus* | 0 | mean, daily max (summer) | 59.322 | 1 | Ettershank (1971) |
| *Iridomyrmex purpureus* | 10 | mean, daily max (summer) | 37.131 | 1 | Ettershank (1971) |
| *Proformica longiseta* | 0 | point sample | 51.637 | 1 | Escudero et al (1997) |
| *Proformica longiseta* | 30 | point sample | 16.98 | 1 | Escudero et al (1997) |

TABLE S2 REFERENCES

Balzani, P., A. Masoni, S. Venturi, F. Frizzi, M. Bambi, R. Fani, B. Nisi, F. Tassi, O. Vaselli, M. Zaccaroni, and G. Santini. 2022. CO2 biogeochemical investigation and microbial characterization of red wood ant mounds in a Southern Europe montane forest. Soil Biology and Biochemistry **166**.

Brandt, D. C. 1980. The thermal diffusivity of the organic material of a mound of *Formica polytena* Foerst. in relation to the thermoregulation of the brood (Hymenoptera, Formicidae). Netherlands Journal of Zoology **30**:326–344.

Bristow, C. M., D. Cappaert, N. J. Campbell, and A. Heise. 1992. Nest structure and colony cycle of the Allegheny mound ant, *Formica exsectoides* Forel (Hymenoptera: Formicidae). Insectes Sociaux **39**:385–402.

Camargo, R., L. C. Forti, C. A. O. Matos, N. Caldato, and O. S. Fonseca. 2016. Is the initial nest depth adapted to favorable conditions for the incipient colony in leaf-cutting ants? Sociobiology **63**:792–799.

Curtis, B. A. 1985. Nests of the Namib desert dune ant *Camponotus detritus* Emery. Insectes Sociaux **32**:313–320.

Escudero, I. F., A. Tinaut, and F. Ruano. 1997. Ovarian maturation under cold winter conditions in a high-mountain ant (Hymenoptera: Formicidae). Environmental Entomology **26**:1373–1377.

Ettershank, G. 1971. Some aspects of the ecology and nest microclimatology of the meat ant, *Iridomyrmex purpureus* (SM.). Proceedings of the Royal Society of Victoria. New Series **84**:137–151.

Rosengren, R., W. Fortelius, K. Lindstrom, and A. Luther. 1987. Phenology and causation of nest heating and thermoregulation in red wood ants of the *Formica rufa* group studied in coniferous forest habitats in southern Finland. Annales Zoologici Fennici **24**:147–155.

Stukalyuk, S., Y. Radchenko, M. Netsvetov, and A. Gilev. 2020. Effect of mound size on intranest thermoregulation in the red wood ants *Formicarufa* and *F. polyctena* (Hymenoptera, Formicidae). Turkish Journal of Zoology **44**:266–280.

**Table S3.** Support for alternative models differing in random effects for probe applied to (a) minimum and (b) maximum daily temperature recorded 1 cm above the nest surface and at a depth of 7 cm inside the nest over 12 weeks. See also Tables 1 and S5. Those models with the lowest AIC value are considered to be the best supported, although any model within 2 AIC units of this model (i.e., ≤2.0 ΔAIC) are equally credible.

|  | model | AIC | ΔAIC |
| --- | --- | --- | --- |
| (a) minimum daily temperatures | | |  |
|  | random intercept + slope | 2480.92 | 6.00 |
|  | random intercept | 2476.92 | 2.00 |
|  | no random effects | 2474.92 | 0.00 |
| (b) maximum daily temperature | | |  |
|  | random intercept + slope | 3152.22 | 4.00 |
|  | random intercept | 3148.22 | 0.00 |
|  | no random effects | 3152.82 | 4.59 |

**Table S4.** Support for alternative models differing in random effects for probe applied to (a) minimum and (b) maximum daily temperature on and off the nest recorded 1 cm above the nest/soil surface versus at a depth of 7 cm over 4 weeks. Those models with the lowest AIC value are the best supported.

|  | model | AIC | | ΔAIC | |
| --- | --- | --- | --- | --- | --- |
| (a) minimum daily temperatures | | |  | |  |
|  | random intercept + slope | 1087.71 | | 4.00 | |
|  | random intercept | 1083.71 | | 0.00 | |
|  | no random effects | 1086.82 | | 3.11 | |
| (b) maximum daily temperature | | |  | |  |
|  | random intercept + slope | 1421.67 | | 3.98 | |
|  | random intercept | 1417.69 | | 0.00 | |
|  | no random effects | 1472.51 | | 54.82 | |

**Table S5.** Differences in minimum daily temperature over 12 weeks recorded 1 cm above the nest surface and at a depth of 7 cm inside the nest based on a model with no random effects (see Table 1 for the equivalent model applied with a random intercept for probe). XI was the most ‘exposed’ nest, XCIII the most ‘sheltered’ nest, and III between the two as the ‘moderate’ nest. K refers to the knot number used for the spline smoothing term associated with daily changes in temperature.

|  | variable | estimate (95% confidence range) | *t* | *F* | *p* |
| --- | --- | --- | --- | --- | --- |
| separate | |  |  |  |  |
|  | intercept (nest III) | 23.22 (22.83, 23.62) | 116.2 |  | < 0.0001 |
|  | nest XCIII | -0.01 (-0.49, 0.47) | -0.03 |  | 0.98 |
|  | nest XI | -0.24 (-0.72, 0.24) | -0.98 |  | 0.33 |
|  | depth (surface vs internal) | -7.23 (-7.63, -6.84) | -36.19 |  | < 0.0001 |
| overall | |  |  |  |  |
|  | nest |  |  | 0.62 | 0.54 |
|  | depth |  |  | 1310.01 | < 0.0001 |
|  | smoothing term (date) |  |  | 165.5 | < 0.0001 |
| *N*_observations, nests, depth_=540, 3, 2 | |  |  |  |  |
| k = 5 | |  |  |  |  |

**Table S6.** Differences in minimum daily temperature on and off the nest recorded 1 cm above the nest/soil surface versus at a depth of 7 cm over 4 weeks. The model applied includes a random intercept for the probe recording data and an interaction for probe depth and location (on versus off the nest). XI was the most ‘exposed’ nest while XCIII was the most ‘sheltered’ nest. See also Table 2.

| variable | estimate (95% confidence range) | *t* | *p* |
| --- | --- | --- | --- |
| intercept (nest XCIII) | 20.37 (19.22, 21.52) | 34.8 | < 0.0001 |
| nest XI | 0.07 (-0.96, 1.09) | 0.13 | 0.90 |
| depth (surface vs internal) | -6.32 (-7.77, -4.87) | -8.53 | < 0.0001 |
| location (on vs off nest) | -0.07 (-1.52, 1.38) | -0.10 | 0.92 |
| depth * location | -0.72 (-2.77, 1.34) | -0.68 | 0.50 |

**Table S7.** Support for alternative OU evolutionary models of brood incubation temperature described as the phylogenetic mean (null model) or as a function of climate zone, material used to construct nests, or their combination (see Table S1). The model with the lowest AIC value is the best supported.

| OU model applied | AIC_c_ | ΔAIC_c_ | N_species_ |
| --- | --- | --- | --- |
| null (intercept only) | 353.6 | 0 | 59 |
| climate zone | 358.5 | 4.9 | 59 |
| nest type | 362.9 | 9.3 | 59 |
| nest type + climate zone | 369.6 | 16 | 59 |

**Figure S1.** Naturally predated meat ant nests by echidna. Excavations into the nest correspond to the position of the brood within the nest at the time of predation. These examples were fresh and likely occurred within one or two weeks of the photograph being taken. Depth was inferred based on a ruler or object of known size being placed in the excavation in an image taken immediately prior, which was then measured using imageJ (imagej.nih.gov/ij) to calculate the depth range in the images shown.
